# Improved antitumor activity against prostate cancer via synergistic targeting of Myc and GFAT-1

**DOI:** 10.7150/thno.76614

**Published:** 2023-01-01

**Authors:** Yue Zhang, Jiang Li, Yixian Huang, Yuang Chen, Zhangyi Luo, Haozhe Huang, Raymond E. West, Thomas D. Nolin, Zhou Wang, Song Li

**Affiliations:** 1 Center for Pharmacogenetics, Department of Pharmaceutical Sciences, University of Pittsburgh School of Pharmacy, Pittsburgh, PA 15261.; 2 Small Molecule Biomarker Core (SMBC), Department of Pharmaceutical Sciences, University of Pittsburgh School of Pharmacy, Pittsburgh, PA 15261.; 3 Department of Urology, University of Pittsburgh School of Medicine, Pittsburgh, PA 15261.

**Keywords:** Myc, glutamine: fructose-6-phosphate amidotransferase-1, protein glycosylation, prostate cancer, therapy

## Abstract

Inhibition of Myc promotes the regression of many types of tumors, including prostate cancer. However, the success of anti-Myc therapy is hampered by the lack of a strategy to effectively deliver the inhibitors to the tumor site and by the feedback mechanisms that cancer cells use to adapt to metabolic reprogramming.

**Methods:** The effects of Myc inhibitors (10074-G5 or 10058-F4), alone or in combination with 6-diazo-5-oxo-L-norleucine (DON), were evaluated in cultured human or murine prostate cancer cells by cell viability assay, qRT-PCR and Western blot. To facilitate the *in vivo* therapeutic evaluation, a prodrug conjugate of 10074-G4 and DON (10074-DON) was developed, which could be effectively loaded into a polysaccharide-based nanocarrier (PS).

**Results:** The treatment with Myc inhibitors led to significant induction of glutamine: fructose-6-phosphate amidotransferase-1 (GFAT1) and enhanced protein glycosylation. Mechanistically, Myc inhibition triggered GFAT1 induction through the IREα-Xbp1s pathway. The combination use of Myc inhibitors and GFAT1 inhibitor DON led to a synergistic effect in inhibiting the proliferation and migration of prostate cancer cells. Enhanced *in vivo* delivery of 10074-DON via the PS nanocarrier led to a significant inhibition of tumor growth along with an improvement in tumor immune microenvironment in several PCa animal models.

**Conclusion:** Simultaneous targeting of Myc and GFAT-1 may represent a novel strategy for the treatment of prostate cancer.

## Introduction

MYC is one of the most highly amplified proto-oncogenes in many types of human cancers 1. As a transcriptional factor, MYC functions through heterodimerization with MAX, and the MYC/MAX complex recognizes DNA response elements to activate the transcription of MYC target genes 1-3. MYC is a master regulator of a broad spectrum of genes involved in cell cycle regulation, DNA replication, energy metabolism, biomass accumulation and mitochondrial biogenesis, all of which appear to promote cell proliferation and growth 4-6. Hence, MYC plays a pivotal role in various processes in cancers, including tumorigenesis, metabolism, metastasis, and immune modulation 7-9.

O-linked N-acetylglucosaminylation (O-GlcNAcylation) is a reversible posttranslational modification that regulates the activities of a wide panel of proteins involved in multiple aspects of cell biology, including sensing the availability of energy and coupling metabolic flux to the control of cell proliferation 10-12. Hyper-O-GlcNAcylation has been shown to play a significant role in cancer development through different mechanisms 13. For instance, the accumulation of O-GlcNAc-modified proteins could induce the proliferation of tumor cells and facilitate immune escape 14,15. Hyper-O-GlcNAcylation can also enhance the metabolic change of cancer cells to favor their growth and contribute to NF-κB oncogenic activation 10,11,16.

Glutamine: fructose-6-phosphate amidotransferase-1 (GFAT1) is the rate-limiting enzyme in the hexosamine biosynthetic pathway (HBP), which utilizes glucose, glutamine, acetyl-coenzyme-A, and nucleotide UTP to synthesize UDP-N-acetyl-D-glucosamine (UDP-GlcNAc) 17. UDP-GlcNAc serves as the substrate for O-GlcNAc transferase (OGT), which catalyzes the transfer of the GlcNAc moiety onto the free hydroxyl of serine and threonine residues 18,19. GFAT-1 has been reported to be upregulated in several types of cancer, and the level of GFAT-1 is negatively correlated with prognosis 20-22. GFAT-1 promotes epithelial-to-mesenchymal transition (EMT) and the invasive activity of gastric cancer by inducing the expression of TGF-β. In addition, increased GFAT-1 expression helps to protect tumor cells from immune attack via enhanced protein glycosylation and reduced lymphocyte infiltration 15,23.

Previous studies have revealed an association of high MYC expression with metabolic alterations in cancer cells, including aberrant protein glycosylation 24,25. MYC enhances glucose metabolism by upregulating glucose transporters (e.g., GLUT1) and several other genes involved in the glycolytic pathway, such as hexokinase 2 (HK2) and lactate dehydrogenase A (LDH-A) 26,27. MYC also directly regulates the key enzymes related to O-GlcNAcylation, which increases the partitioning of glucose carbon to HBP at the entrance of the glycolysis pathway 25,28,30.

Here, we report our unexpected observation that both pharmacological and genetic inhibition of Myc in prostate cancer cells led to significant upregulation of the expression level of GFAT-1 and enhanced protein glycosylation. The underlying mechanism of GFAT-1 induction and the synergistic antitumor activity of simultaneous targeting of Myc and GFAT-1 were also investigated. To facilitate the therapeutic application of this novel finding, a prodrug conjugate of 10074-G4 and DON (a GFAT-1 inhibitor) and a polysaccharide-based nanocarrier (PS) were developed to improve drug delivery to tumors. The antitumor activity of 10074-DON-loaded PS nanoparticles was investigated in 3 prostate cancer models. The impact of this treatment on the tumor immune microenvironment was also studied.

## Results

### Inhibition of Myc leads to an increase in GFAT-1 expression and protein glycosylation in PC3 and RM1 cell lines

**Figure 1:**
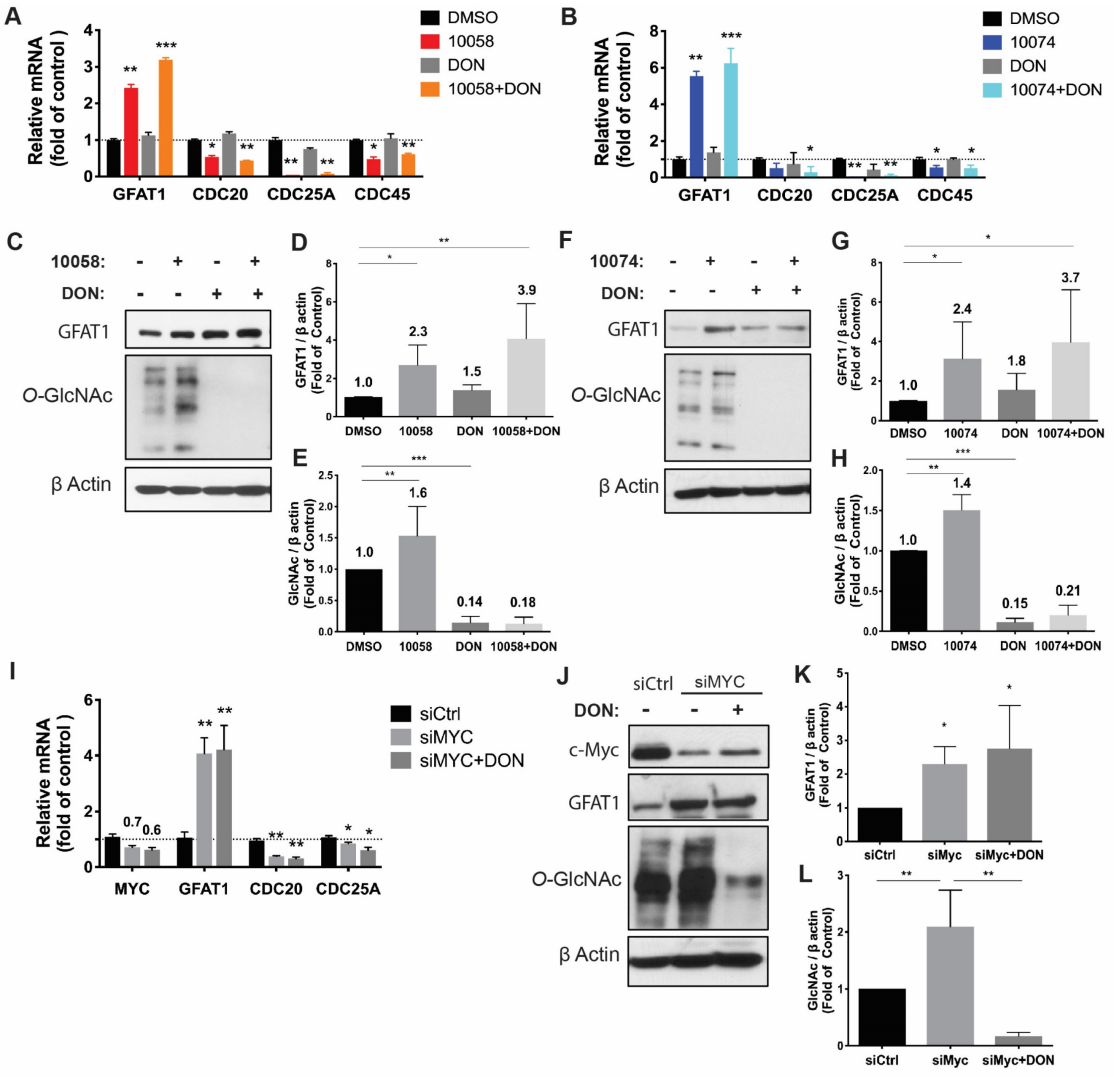
**Inhibition of Myc leads to GFAT1 upregulation and increased total protein glycosylation in PC3 cells**: Human prostate cancer PC3 cells were treated with 25 μM 10058-F4 (10058) or 10074-G5 (10074), 25 μM DON, or a combination of DON with 10058 (10078) for 24 hours. (**A-B**) Total RNA from PC3 cells was purified for qRT-PCR analysis. (**C**) Western blot assay of total proteins from PC3 cells treated with 10058. (**D-E**) Quantification of the Western blot results from (**C**) by ImageJ. (**F**) Western blot assay of total proteins from PC3 cells treated with 10074. (**G-H**) Quantification of the Western blot results from (**F**) by ImageJ. (**I-J**) RT-PCR (**I**) and Western blot (**J**) analysis of GFAT1 expression in PC3 cells treated with siMyc. (**K-L**) Quantification of the Western blot results from (**J**). The results are expressed as the mean ± SEM of triplicate measurements in each group. *p < 0.05, **p < 0.01, ***p < 0.001.

To study the effect of Myc inhibition on glucose metabolism in prostate cancer cells, we first examined the effect of Myc inhibitors on the mRNA expression levels of several genes critically involved in glucose metabolism, including glucose transporter 1 (GLUT1), glutaminase (GLS), hexokinase 2 (HK2), and lactate dehydrogenase A (LDHA). 10058 and 10074 are Myc inhibitors that work by disrupting Myc/Max dimerization, and the activity of the two compounds in the PC3 human prostate cancer cell line was demonstrated by the significant inhibition of the mRNA expression of three (3) Myc target genes, CDC20, CDC25A, and CDC45 (**Figure 1A**). **Figure S1A** shows that the mRNA levels of GLUT1 and LDHA were downregulated by both 10058 and 10074, consistent with the role of Myc in promoting glycolysis in tumor cells. The mRNA levels of GLS and HK2 did not show significant changes following treatment with either Myc inhibitor. Interestingly, among all genes studied, GFAT-1 was the only gene that was significantly upregulated by Myc inhibition (**Figure S1A** and **Figure 1A**). The effect of Myc inhibition on GFAT-1 expression was further confirmed with Western blotting (**Figure 1C, D, F, and G**). Both 10058 and 10074 significantly increased GFAT1 expression at the protein level (**Figure 1C-G**).

GFAT1 is the rate-limiting enzyme that catalyzes the synthesis of UDP-GlcNAc, a substrate for protein glycosylation. Therefore, the level of protein glycosylation was examined by Western blot after treatment with 10078 or 10058. **Figure 1C and E** show that upregulation of GFAT-1 was associated with a significant increase in protein glycosylation following treatment with 10074. Similar results were shown following treatment with 10058 (**Figure 1F and H, Figure S1B-D**).

Diazo-5-oxo-L-norleucine (DON) is an inhibitor of GFAT, and treatment with DON significantly downregulated both basal and 10058/10074-induced increases in protein glycosylation (**Figure 1C, E, F, and H**). Interestingly, DON also caused slight increases in the expression of GFAT-1 at both the mRNA and protein levels (**Figure 1A-D, F and G**). However, DON had no effects on the expression of other Myc target genes. It is likely that DON induced GFAT-1 expression through a Myc-independent feedback mechanism.

To confirm that the effect of 10058 and 10074 on GFAT1 upregulation was Myc inhibition-specific, the above experiment was similarly performed in PC3 cells in which Myc expression was knocked down by Myc-specific siRNA (siMyc). Myc knockdown was confirmed at both the mRNA and protein levels (**Figure 1I-J**). **Fig 1I~K** shows that GFAT-1 was similarly upregulated at both the mRNA and protein levels following siMyc treatment. SiMyc treatment also led to an increase in the level of protein glycosylation (**Figure 1J and L**), supporting the notion that Myc inhibition causes increased protein glycosylation through GFAT-1 induction. The Myc inhibition did not affect the protein expression level of O-GlcNAc Transferase1 (OGT1), another enzyme critically involved in O-GlcNAcylation (**Figure S1E**). In addition, Myc inhibition had no impact on the phosphorylation of GFAT1 (**Figure S1F**).

**Figure 2:**
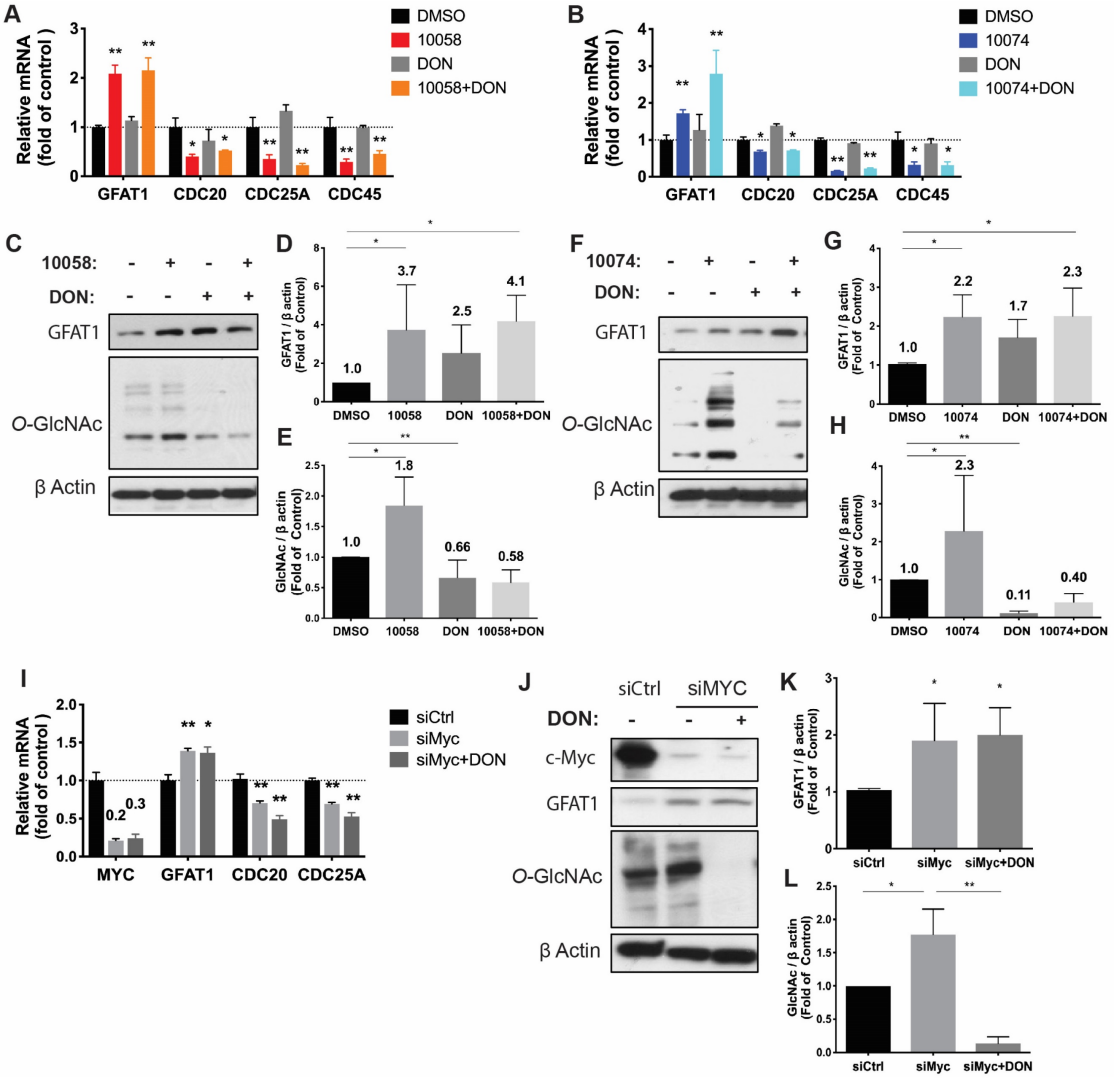
** Inhibition of Myc leads to GFAT1 upregulation and increased total protein glycosylation in RM-1 cells**: Murine prostate cancer RM-1 cells were treated with 25 μM 10058 or 10074, 25 μM DON, or a combination of DON and 10058 (10078) for 24 hours. (**A-B**) Total RNA of RM1 was purified for qPCR analysis. (**C**) Total proteins were extracted from RM-1 cells treated with 10058 for Western blot assays. (**D-E**) Quantification of the Western blot results from (**C**) by ImageJ. (**F**) Total proteins were extracted from RM-1 cells treated with 10074 for Western blot assays. (**G-H**) Quantification of the Western blot results from (**D**) by ImageJ. (**I-J**) RT-PCR (**I**) and Western blot (**J**) analysis of GFAT1 expression in RM-1 cells treated with siMyc. (**K-L**) Quantification of the Western blot results from (**J**). The results are expressed as the mean ± SEM of triplicate measurements in each group. *p < 0.05, **p < 0.01, ***p < 0.001.

Similar results were observed in RM-1, a murine prostate cancer cell line that was generated from a Ras+Myc-induced prostate cancer that developed from a urogenital sinus mouse prostate reconstitution in male C57BL/6 mice (**Figure 2**). Both pharmacological (10078 or 10058) and genetic (siMyc) inhibition of Myc led to GFAT-1 upregulation and increased protein glycosylation (**Figure 2**). These findings were further confirmed in Myc-Cap, a cell line derived from spontaneous prostate cancer in c-Myc transgenic mice (**Figure S2**).

### Synergistic Effect of Myc and GFAT1 inhibitors

**Figure 3:**
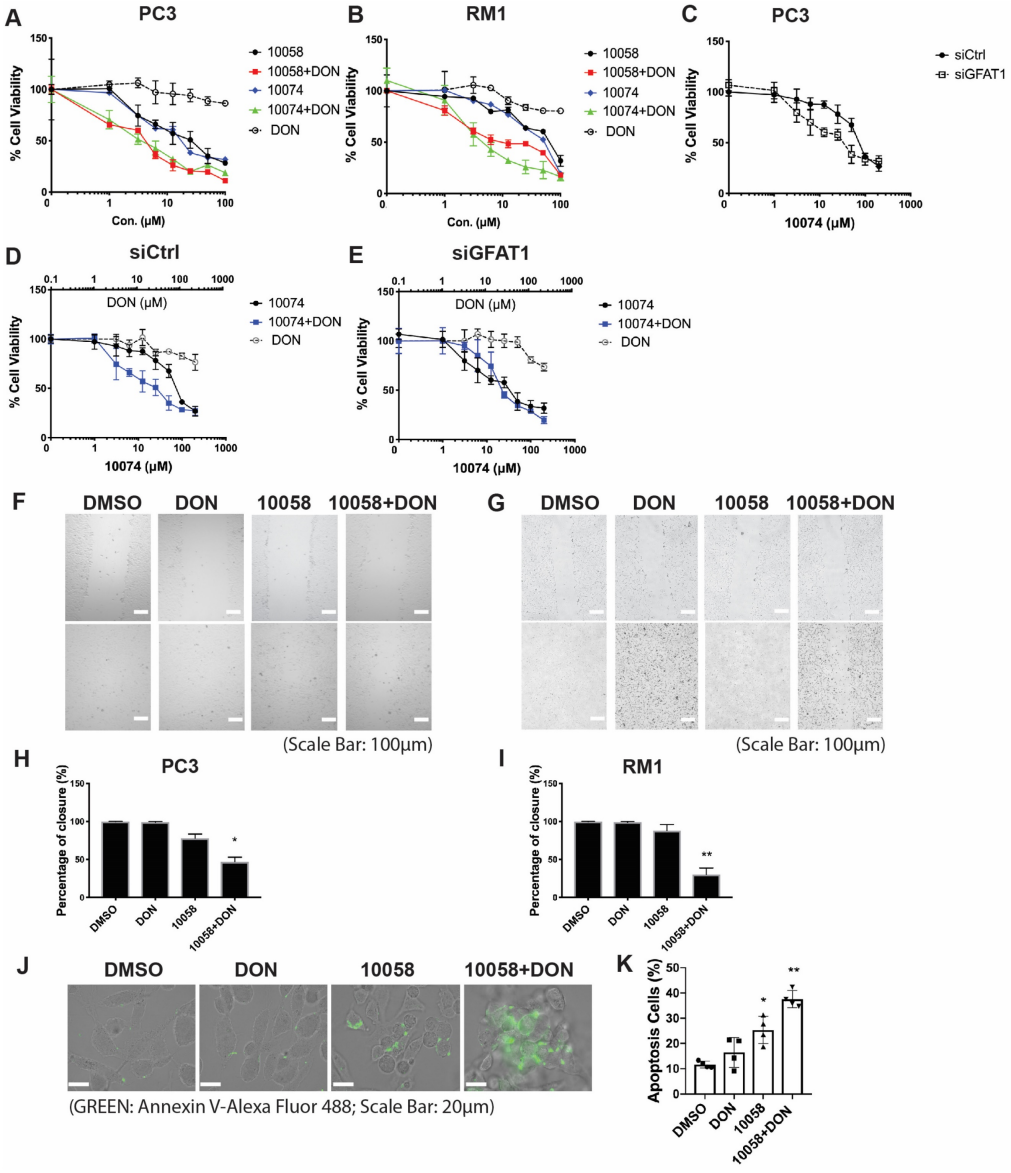
** Myc and GFAT1 inhibitors have a synergistic effect in inhibiting prostate cancer cell proliferation and migration while inducing apoptosis: (A-B)** Treatment with DON enhanced the cytotoxicity of 10058 or 10074 in PC3 (A) and RM-1 (B) cells in the MTT assay. (C) MTT assay of the cytotoxicity of 10058 or 10074 in PC cells treated with control siRNA or siGFAT1. **(D-E)** Knockdown of GFAT1 enhanced the sensitivity of PC3 cells to 10074 treatment, mimicking the effect of DON. **(F-I)** The combination of 10058 with DON significantly reduced the migration of PC3 (F) and RM-1 (G) cells in a wound-healing assay. **(J-K)** The combination of 10058 with DON significantly increased the percentages of apoptotic cells compared to single treatment. The results are expressed as the mean ± SEM of triplicate measurements in each group. *p < 0.05, **p < 0.01, ***p < 0.001.

Upregulation of GFAT-1 and enhanced protein glycosylation have been implicated in facilitating tumor cell proliferation and cell migration (20-24). We hypothesized that simultaneous inhibition of Myc and GFAT-1 would result in enhanced cytotoxicity towards cancer cells. To test this hypothesis, we examined the cytotoxicity following treatment with 10058 or 10074 alone and in combination with DON via MTT assay. DON alone showed minimal effect on the proliferation of PC3 cancer cells at the doses tested (**Figure 3A**). Compound 10058 or 10074 alone inhibited the proliferation of PC3 cells in a concentration-dependent manner, while the combination of DON with either inhibitor led to a significant increase in the level of cytotoxicity (**Figure 3A**). Similar results were observed in the RM-1 cell line (**Figure 3B**) and Myc-Cap cell line (**Figure S3**). In addition, knockdown of GFAT1 by siRNA enhanced the sensitivity of PC3 cells to 10074, mimicking the effect of DON (**Figure 3C**). **Figure 3D-E** shows that the enhanced cytotoxicity of the 10074 and DON combination was significantly attenuated in PC3 cells treated with siGFAT1 compared to cells treated with control siRNA, suggesting that the synergistic effect of the Myc inhibitor and DON is GFAT1-dependent.

We further evaluated the synergistic effect of Myc and GFAT inhibitors on cancer cell migration by a wound healing assay. A monolayer of PC3 cells was intentionally streaked to generate a wound in cell culture. After 24 hours, the wounded area was substantially repopulated by tumor cells in the control group that received no treatment (**Figure 3F and H**). Treatment with 10058 alone had a slight effect on inhibiting the migration of cancer cells, while DON showed no effect (**Figure 3F and H**). In contrast, the combination of both led to significant improvement in inhibiting the migration of PC3 cells; the denuded area was well retained with only 47% wound closure (**Figure 3F and H**). Similar results were shown in RM-1 cells (**Figure 3G and I**). Annexin V staining further showed that combination treatment with 10058 and DON led to a significant increase in the number of apoptotic PC3 cells (**Figure 3J-K**). Taken together, the simultaneous inhibition of Myc and GFAT-1 exhibited a synergistic effect in the inhibition of both the proliferation and migration of prostate cancer cells while inducing more cancer cell apoptosis.

### Inhibition of Myc leads to GFAT-1 upregulation through modulation of ER stress and the transcriptional factor Xbp1-spliced form

**Figure 4:**
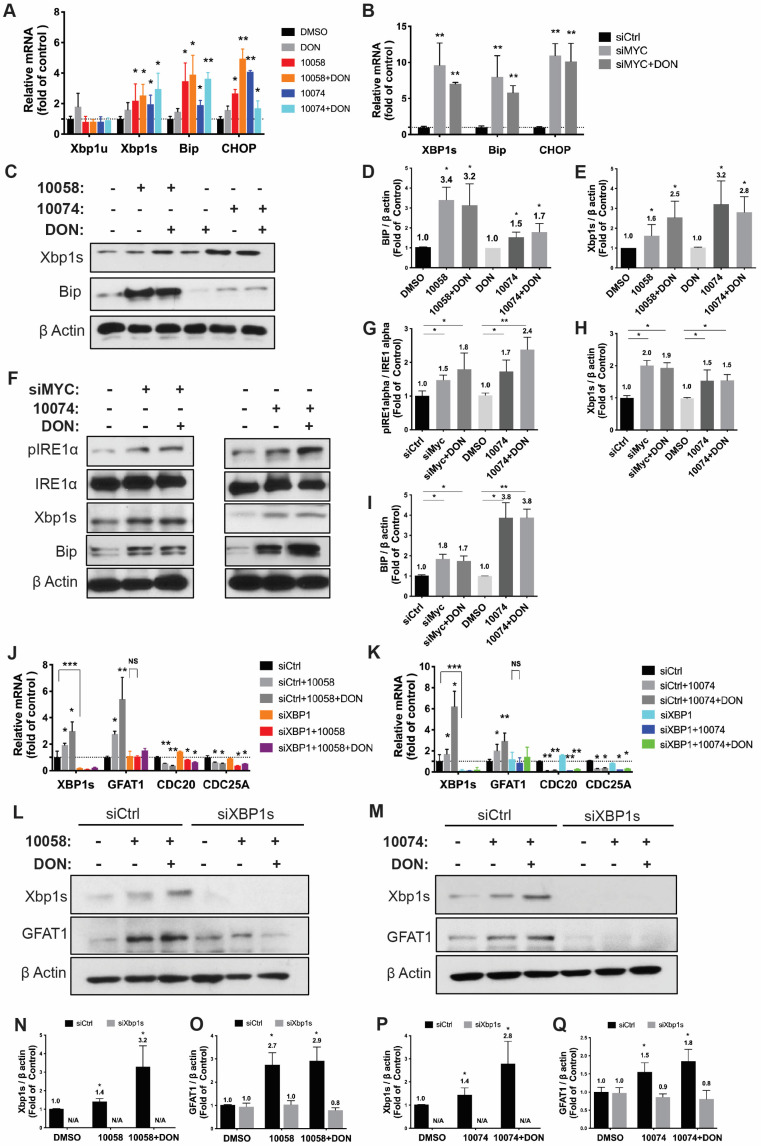
** Inhibition of Myc leads to GFAT1 upregulation through induction of ER stress and the transcriptional factor Xbp1-spliced form: (A)** Real-time PCR analysis revealed that the Myc inhibitor 10058 or 10074 upregulated the mRNA level of the xbp1 spliced form (xbp1s) but not the unspliced form (xbp1u). 10058 or 10074 also upregulated BIP and CHOP, the target genes of xbp1s. **(B)** Knockdown of Myc by siRNA upregulated the mRNA levels of xbp1s, BIP, and CHOP. **(C-E)** Bip protein was upregulated by both 10058 and 10074, as shown by Western blot. **(F-I)** The Myc inhibitor 10074 or siMyc did not change the protein level of IRE1α but increased the level of phosphorylated IRE1α. **(J-M)** Knockdown of xbp1s by siRNA abolished the effect of 10058 (J, L) and 10074 (K, M) in upregulating the expression of GFAT1 at both the mRNA and protein levels. **(N-Q)** Quantification of western results from (L-M). The results are expressed as the mean ± SEM of triplicate measurements in each group. *p < 0.05, **p < 0.01, ***p < 0.001.

It has been reported that GFAT-1 is induced in heart ischemia via the XBP1/GFAT-1 pathway 31. To examine whether XBP-1 is similarly involved in GFAT-1 induction following Myc inhibition, we first examined the effect of Myc inhibitors on the expression levels of XBP1. Interestingly, we found that both 10058 and 10074 could induce upregulation of the mRNA of the XBP1 spliced form (xbp1s) but not the un-spliced form (xbp1u) (**Figure 4A**). Classical XBP1s target genes, such as BIP and CHOP, were also significantly induced following treatment with 10058 or 10074 (**Figure 4A, 4C-E**). These two genes are known to be robustly induced during ER stress, suggesting that Myc inhibition causes ER stress, resulting in the induction of XBP-1. Knocking down Myc by siRNA also induced xbp1s, BIP and CHOP (**Figure 4B**), further suggesting that XBP-1 induction is specifically mediated by Myc inhibition. Since XBP1 mRNA is spliced by serine/threonine-protein kinase/endoribonuclease inositol-requiring enzyme 1 α (IRE1α) 32, we examined the protein levels of IRE1α and its phosphorylated form (pIRE1α) after Myc inhibition. **Figure 4F** shows that both 10074 and Myc siRNA had no effect on the protein level of IRE1α but significantly increased the level of pIRE1α, indicating that Myc inhibition induces XBP1s upregulation through increased IRE1 activity (**Figure 4G-I**). Using classical ER stress inducer 1,4-Dithiothreitol (DTT), we were also able to induce up-regulation of BIP, Xbp1s and GFAT1 at protein level (**Figure S4**), further supporting our hypothesis that Inhibition of Myc leads to GFAT-1 upregulation through modulation of ER stress.

To further establish a role for XBP1s in Myc inhibition-mediated GFAT-1 induction, we knocked down XBP1 expression in PC-3 cells via RNAi and then similarly determined the expression level of GFAT-1 after treatment with 10058 or 10074. As shown in **Figure 4J and 4K**, knockdown of XBP1 via siRNA significantly attenuated the 10058-induced GFAT-1 upregulation at both the mRNA and protein levels while showing no effect on other Myc target genes. Similar results were shown in PC3 cells treated with XBP-1 siRNA followed by 10074 treatment (**Figure 4N-Q**), suggesting a role of the XBP1s/GFAT-1 pathway in Myc inhibition-induced GFAT-1 expression. In addition, knockdown of GFAT1 by siRNA abolished the effect of 10074 in upregulating GFAT1 at both the mRNA and protein levels but did not affect the expression levels of Xbp1s and other ER stress markers, such as Bip (**Figure S5**), suggesting that GFAT1 is located downstream of the IRE1/XBP1 signaling pathway.

### Characterization of the prodrug 10074-DON

**Figure 5:**
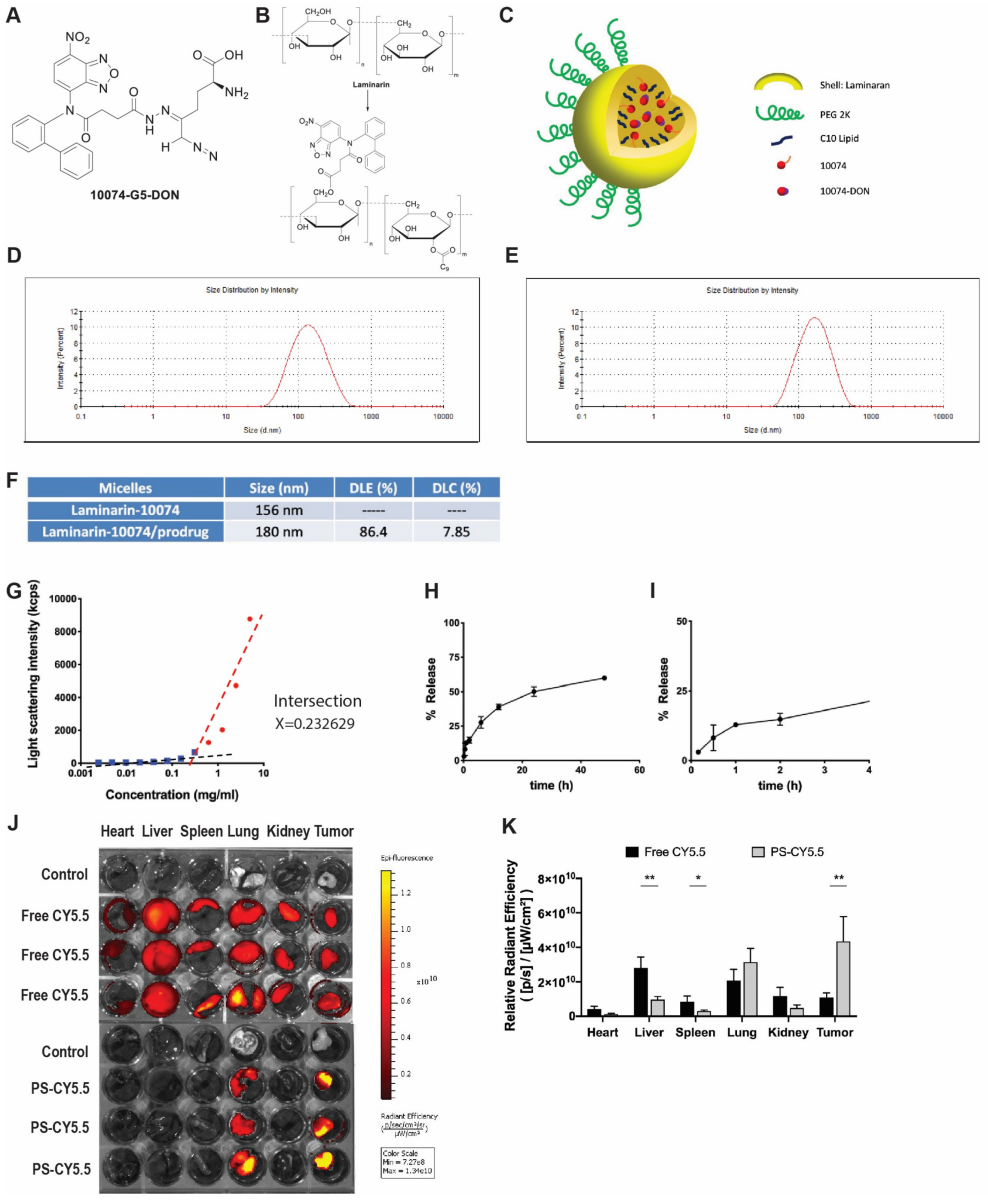
**Characterization of the 10074-DON prodrug and the PS nanocarrier system**: (**A**) Chemical structure of the 10074-DON conjugate. (**B**) Structures of laminarin and 10074-derivatized laminarin (PS nanocarrier). **(C)** A carton showing the self-assembly of PS nanocarriers loaded with 10074 or 10074-DON. (**D-E**) DLS analysis of blank PS micelles and PS micelles loaded with 10074-DON prodrug. (**F**) DLC and DLE of 10074-DON in 10074-DON-loaded PS micelles. (**G**) CMC of 10074-derivatized laminarin. (**H-I**) The profile of release of 10074-DON prodrug from 10074-DON-loaded PS micelles in 48 hours (**H**) and 4 hours (**I**) (**J-K**) The biodistribution of nanocarrier-loaded fluorescent dye CY5.5 in major organs. The results are expressed as the mean ± SEM of triplicate samples in each group. *p < 0.05, **p < 0.01.

**Scheme 1:**
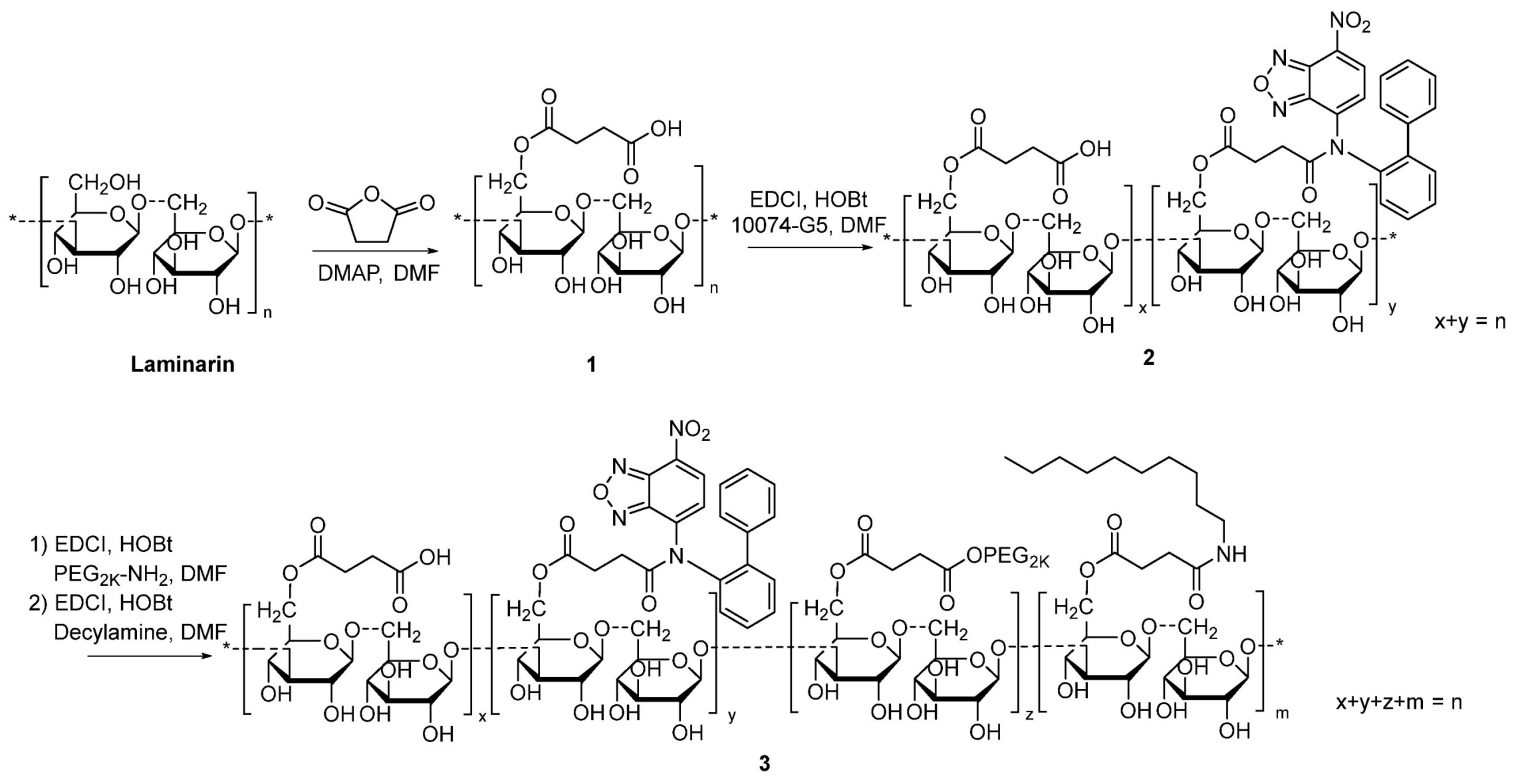
Synthesis of 10074-laminarin conjugate.

**Scheme 2:**
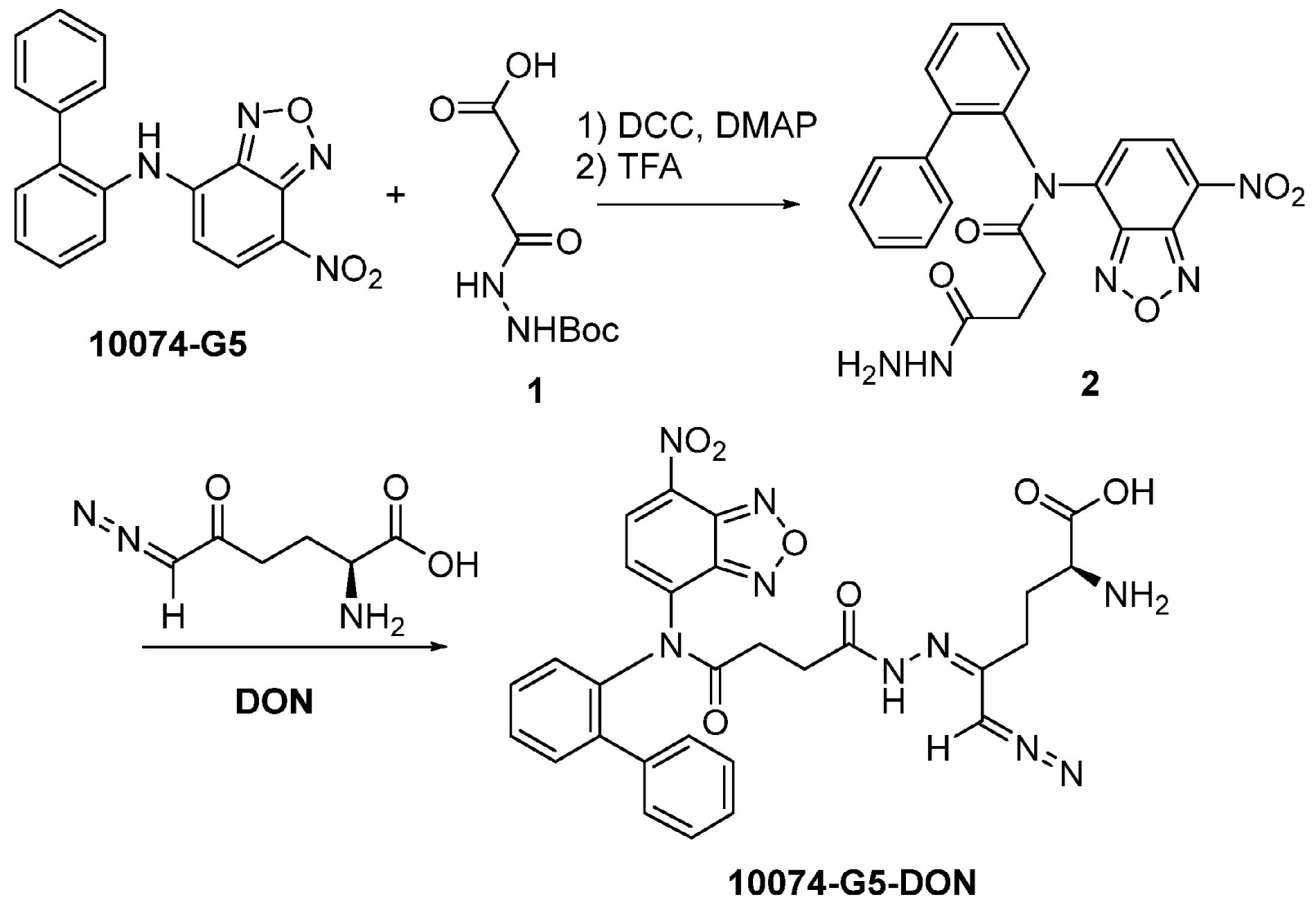
Synthesis of 10074-DON conjugate.

The above studies suggest that combining a Myc inhibitor such as 10074 or 10058 and a GFAT-1 inhibitor such as DON may represent a promising strategy to improve the treatment of prostate cancer. Despite the reported specificity and efficacy of 10074 or 10058 in cultured tumor cells, the *in vivo* application is limited by poor stability, resulting in disappointed antitumor activity *in vivo* 3. On the other hand, DON also has limited bioavailability. In addition, DON may be associated with gastroenteric toxicity 33. Nanocarriers are known to improve selective drug delivery to tumors while minimizing systemic toxicity 34. However, codelivery of 10074 and DON has proven to be challenging, as 10074 is poorly water soluble, while DON is highly water soluble. To develop a strategy for the *in vivo* codelivery of 10074 and DON, a prodrug conjugate of 10074-DON was developed (**Figure 5A**). 10074-DON has an overall hydrophobic nature and could be loaded into an amphiphilic micellar carrier based on 10074-conjugated laminarin (**Figure 5B-C**). The polysaccharide (PS) laminarin is a water-soluble macromolecule and was rendered amphiphilic following the introduction of a small amount of 10074 (**Figure 5B**). 10074 was also introduced into laminarin to facilitate the interaction between the 10074-derivatized drug carrier (PS) and the loaded drug (10074-DON) (**Figure 5A-C**) based on the principle of “like dissolves like” 35,36. The synthesis routes for the PS carrier and 10074-DON are shown in **Schemes 1 and 2**, respectively, and their chemical characterizations (NMR) are shown in **Figure S6**. Both 10074 and 10074-DON could be readily loaded into PS nanocarriers with nanoparticle sizes of approximately 150-180 nm (**Figure 5D-E**). The drug loading capacity (DLC) and drug loading efficiency (DLE) of 10074-DON prodrug were shown in **Figure 5F**. **Figure 5G-I** show the CMC of 10074-derivatized laminarin and the profile of release of 10074-DON prodrug from 10074-DON-loaded PS micelles in 4hours and 48 hours.

Next, we evaluated the biodistribution of polysaccharide (PS) nanocarrier in the RM-1 syngeneic model. PS was conjugated with fluorescent dye CY5.5 (PS-CY5.5) to follow the carrier's distribution in RM1 tumor-bearing mice, with mice treated with equal amount of free CY5.5 dye as control. **Figure 5J** shows the *ex vivo* imaging of the major organs harvested at 24 h post-injection. More CY5.5 signals were observed in the tumor tissue from mice injected with PS-CY5.5 compared to tumors from mice treated with free CY5.5. In addition, less signals of CY5.5 were observed in other major organs, including livers and spleens from mice treated with PS-CY5.5 (**Figure 5K**). These results indicated that PS carrier was highly effective in mediating selective accumulation in the prostate cancer model.

**Figure 6:**
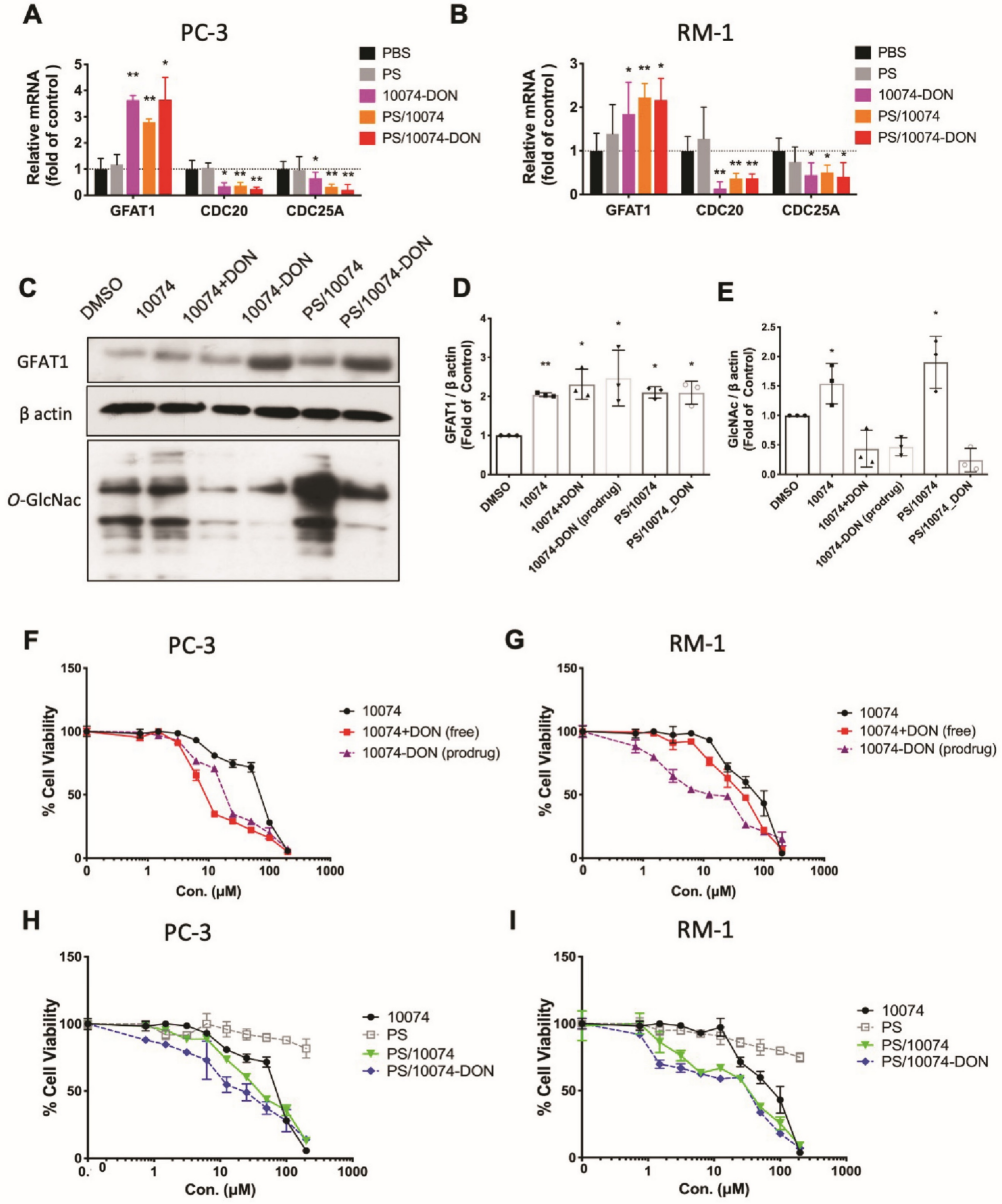
**Biophysical characterizations of PS nanocarrier loaded with 10074-DON prodrug. (A-B)** Real-time PCR analysis revealed that 10074-DON prodrug, PS/10074 or PS/10074-DON retained the ability of 10074 to upregulate the mRNA level of GFAT1. **(C-E)** Western blot analysis of RM-1 cell lysate revealed that 10074-DON prodrug retained the ability of 10074 to upregulate GFAT1 and protein glycosylation. **(F-I)** The 10074-DON prodrug worked more efficiently in inhibiting tumor cell proliferation than 10074 alone in PC3 (**F, H**) and RM-1 cells (**G, I**) as shown in the MTT assay. The results are expressed as the mean ± SEM of triplicate measurements in each group. *p < 0.05, **p < 0.01, ***p < 0.001.

Real-time PCR and Western blotting showed that the prodrug 10074-DON retained the ability of 10074 to induce GFAT1 expression and the effect of DON in suppressing glycosylation (**Figure 6A-E**), indicating that 10074-DON could successfully release both compounds following intracellular delivery. **Figure 6F-G** show that 10074-DON prodrug was slightly less cytotoxic to PC3 cells than the free 10074/DON combination but was more active than 10074/DON in RM1 cells. The improved cytotoxicity of the conjugate over the free combination in RM1 cells might be attributed to improved uptake of the two drugs following covalent conjugation. **Figure S5H-I** shows that 10074 loaded into the PS nanocarrier was slightly more active than free 10074 in both PC3 and RM1 cells, while PS/10074-DON was more active than PS/10074 at low concentrations. The PS carrier alone showed minimal cytotoxicity even at the highest concentration tested (200 µM), suggesting that laminarin is safe and capable of effectively mediating the intracellular delivery of 10074 or 10074-DON.

### Improved *in vivo* antitumor activity of 10074-DON loaded into PS nanocarrier

**Figure 7:**
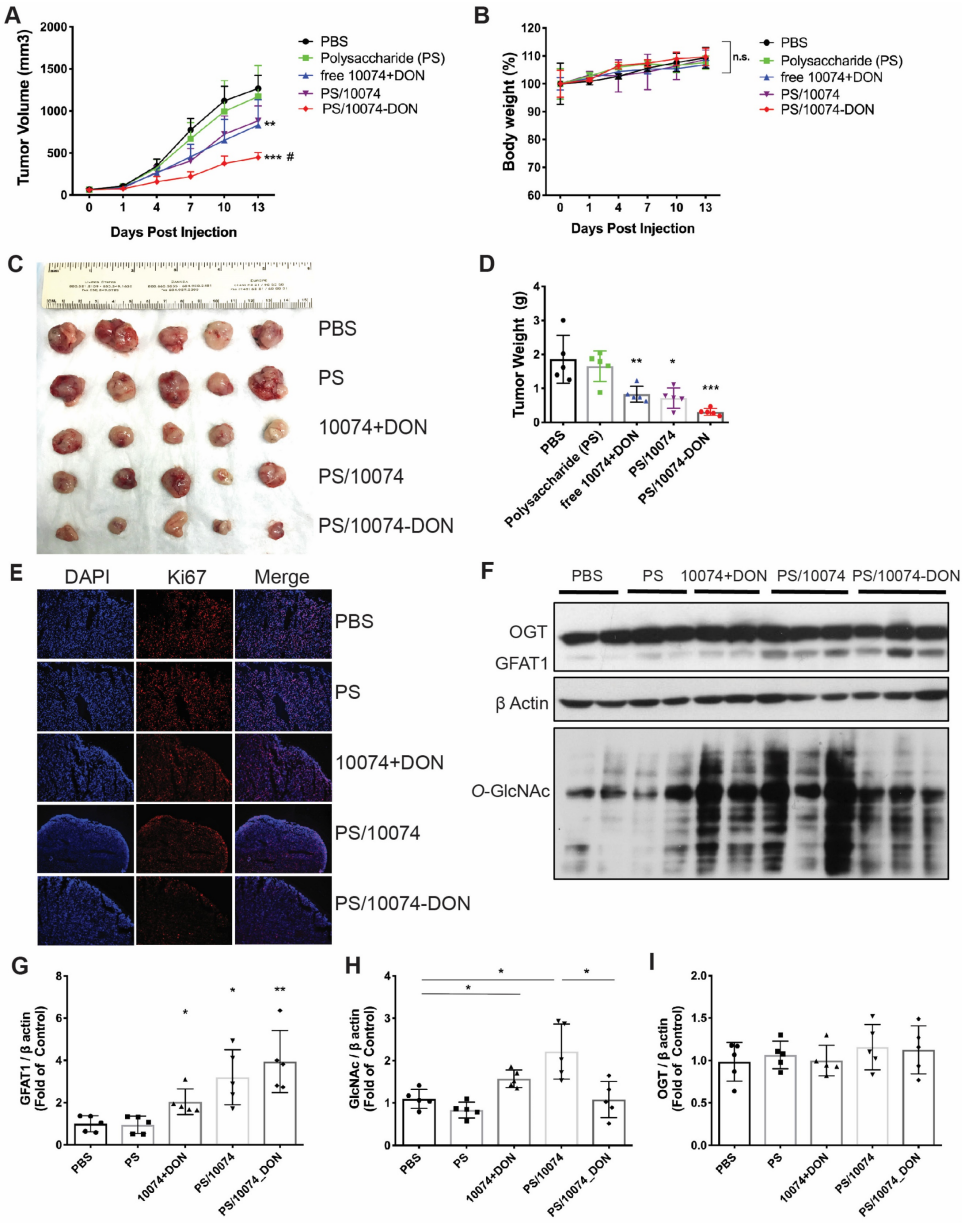
***In vivo* antitumor effect of PS nanocarriers loaded with 10074-DON in the RM-1 syngeneic tumor model**: (**A**) Tumor growth curves in the RM-1 xenograft model. (**B**) Body weight changes during the treatment. (**C**) Gross images of endpoint tumors. (**D**) Endpoint tumor weights in the RM-1 tumor model. (**E**) Ki67 immunostaining of tumor tissue sections. (**F**) Western blot analysis of the tumor tissues collected in (**C**) and the quantification by ImageJ (**G-I**). The results are expressed as the mean ± SEM of measurements from 4 ~ 6 animals per group, and each dot represents a measurement from one animal. *p < 0.05, **p < 0.01, ***p < 0.001.

Following demonstration of effective intracellular delivery of 10074-DON conjugate by PS nanocarrier, the *in vivo* antitumor activity of PS/10074-DON was further evaluated in 3 prostate cancer models. **Figure 6** shows the results in the RM-1 murine prostate cancer model. Groups of 5 mice were inoculated subcutaneously (s.c.). with RM-1 cells, and treatments were started when the tumors reached ~50 mm^3^ in size. Mice received intravenous injection (i.v.) of PBS, PS carrier, free 10074/DON combination, 10074-loaded PS NPs, or 10074-DON-loaded PS NPs once every 3 days 5 times. The PS carrier alone showed no effect on tumor growth. The free 10074/DON combination and PS/10074 showed comparable but only moderate effects in suppressing RM-1 tumor growth, while PS/10074-DON showed the most drastic antitumor effect *in vivo* (**Figure 7A**). All treatments were well tolerated, as evident from the normal gains in body weight (**Figure 7B**). A similar conclusion was reached by measuring the endpoint tumor sizes and weights (**Figure 7C-D**). Staining of the frozen tumor sections with ki67 antibody indicated that PS/10074-DON significantly reduced the proliferation of tumor cells (**Figure 7E**).

Western blot analysis of tumor tissues showed that all groups receiving 10074 treatment had significant increases in the levels of GFAT1 expression and total protein glycosylation (**Figure 7F-H**). Tumors treated with PS/10074-DON maintained a relatively lower level of protein glycosylation than PS/10074-treated tumors, indicating that the prodrug could release DON to suppress glycosylation *in vivo* (**Figure 7F and H**). No differences were found among the groups in the protein level of *O*-GlcNac transferase (OGT) (**Figure 7I**), indicating that changes in glycosylation were primarily attributed to the different levels of GFAT1.

**Figure 8:**
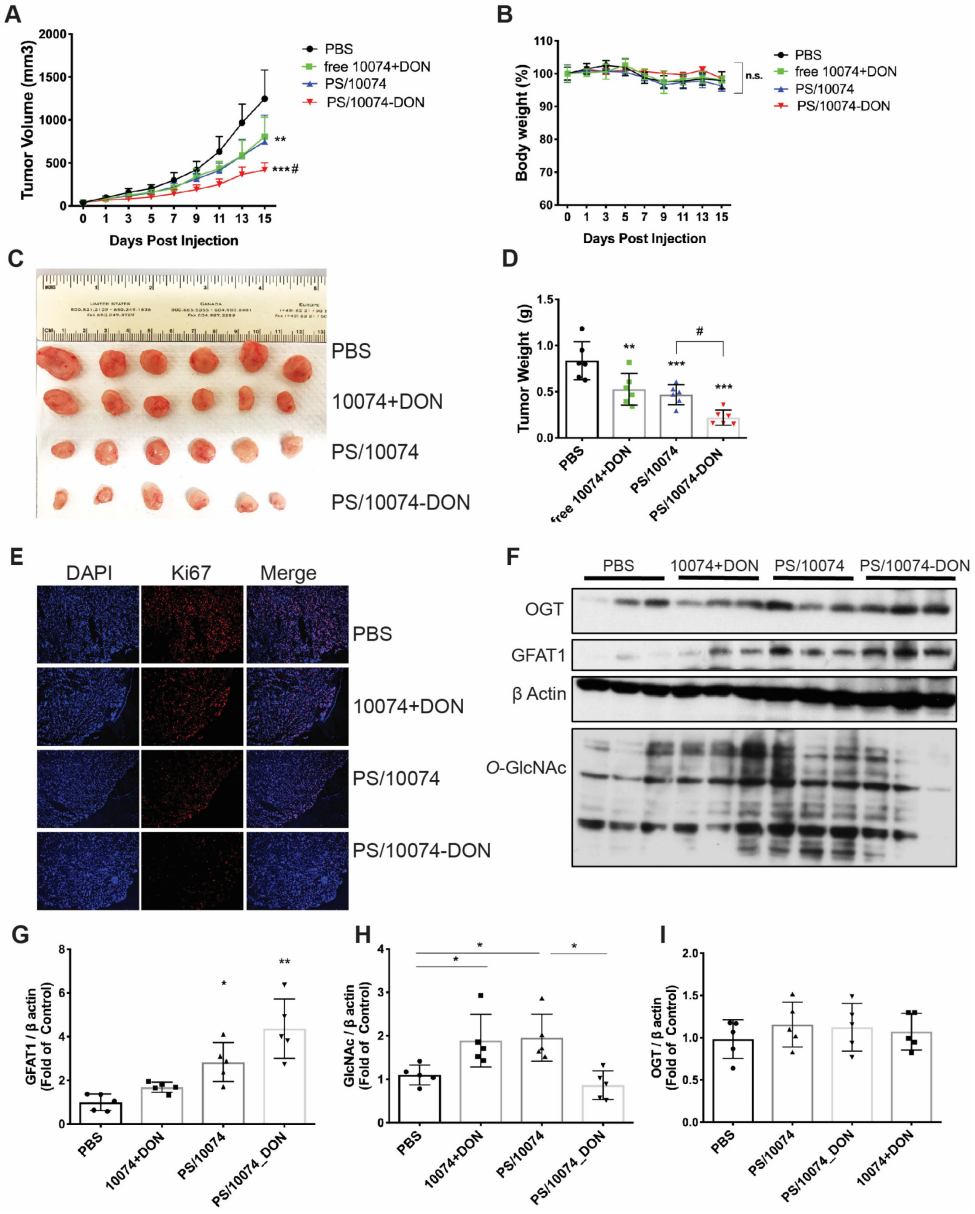
***In vivo* antitumor effect of PS nanocarriers loaded with 10074-DON in the PC3 tumor model**: (**A**) Tumor growth curves in the PC3 xenograft model. (**B**) Body weight changes during the treatment. (**C**) Gross images of endpoint tumors. (**D**) Endpoint tumor weights in the RM-1 xenograft model. (**E**) Ki67 immunostaining of tumor tissue sections. (**F**) Western blot analysis of the tumor tissues collected in (**C**) and the quantification by ImageJ (**G-I**). The results are expressed as the mean ± SEM of measurements from 6 mice per group, and each dot represents a measurement from one animal. *p < 0.05, **p < 0.01, ***p < 0.001.

The *in vivo* antitumor effect of 10074-DON/PS was further evaluated in a PC3 human prostate cancer model using immunodeficient NU/J mice. Consistent with the data in the RM-1 tumor model, we also observed significantly improved antitumor activity for PS/10074-DON in the PC-3 model (**Figure 8A-E**). The prodrug-loaded NPs significantly inhibited tumor growth and decreased the levels of protein glycosylation (**Figure 8F-I**). Similar results were observed in the Myc-Cap murine prostate cancer model using FVB mice (**Figure S7**).

### Flow cytometry analysis of immune cell subsets in tumor tissues

**Figure 9:**
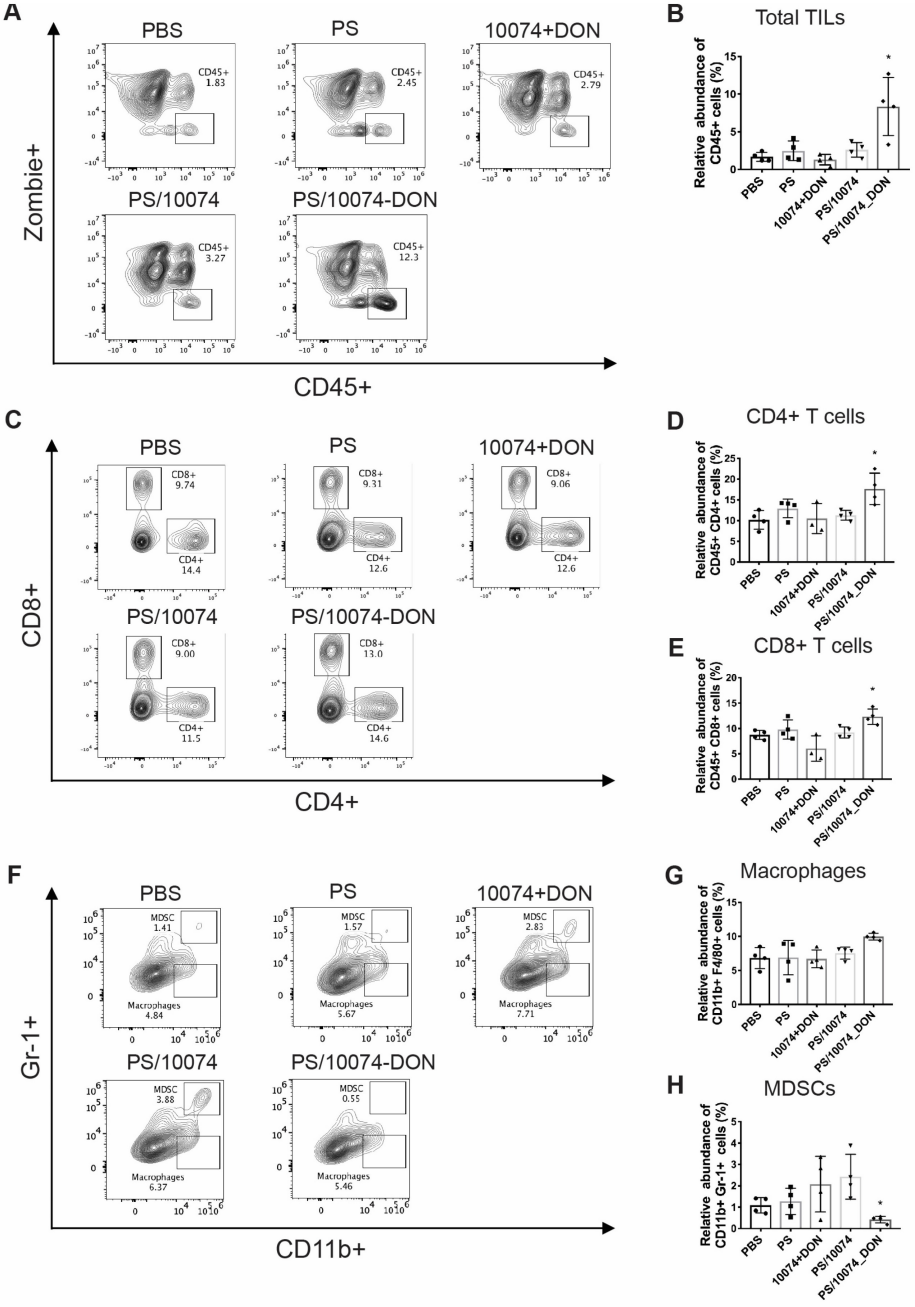
**Flow cytometry analysis of immune cell subsets in RM-1 tumor tissues with various treatments**: (**A-B**) Total immune cell infiltration in RM-1 tumors treated with PS carrier alone, free 10074/DON combination, PS/10074 or PS/10074-DON at a 10074 dosage of 10 mg/kg. (**C-E**) The relative abundance of CD4^+^ and CD8^+^ T cells in tumor tissues. (**F-H**) Flow cytometry gating and histograms of macrophages and CD11b^+^/Gr-1^+^ MDSCs in mouse tumors. The results are expressed as the mean ± SEM of measurements from 4 animals per group, and each dot represents a measurement from one animal. *p < 0.05, **p < 0.01, ***p < 0.001.

Inhibition of Myc or GFAT-1 has been shown to improve the tumor immune microenvironment in addition to having a direct effect on tumor cells 37-39. To delineate the role of the immune response in the improved antitumor activity of PS/10074-DON, the immune cell populations in the tumor tissues with various treatments were analyzed by flow cytometry one day following five injections. As shown in **Figure 9A-B**, tumors treated with PS/10074-DON had significantly more infiltration of CD45^+^ immune cells compared with the control or PS/10074 groups. There were also more CD4^+^ and CD8^+^ T cells in the tumor tissues treated with PS/10074-DON than in the other groups (**Figure 9C-E**). Consistent with the data from the flow study, we also observed increases in CD45^+^ and CD8^+^ cells in the PS/10074-DON group in immunostaining of frozen tumor sections (**Figure S8**).

The infiltrated macrophage (CD11b^+^/F4/80^+^) population was similar among all treatment groups (**Figure 9G**). However, the numbers of myeloid-derived suppressor cells (MDSCs, Gr-1^+^/CD11b^+^) were significantly decreased in the tumors treated with PS/10074-DON (**Figure 9F**).

**Figure 10:**
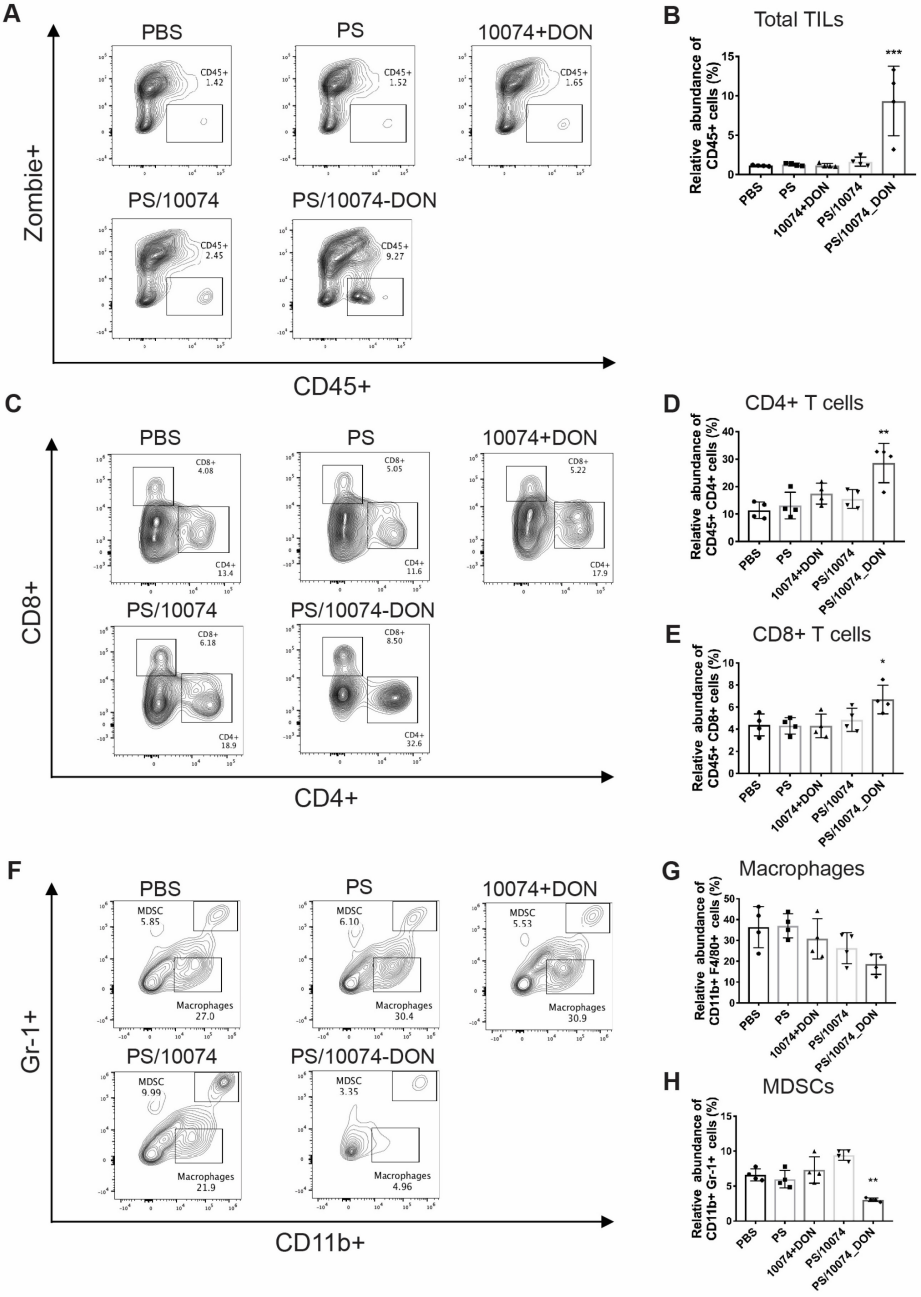
**Flow cytometry analysis of immune cell subsets in Myc-CaP tumor tissues with various treatments**: (**A-B**) Total lymphocyte infiltration in Myc-Cap tumors treated with PS carrier alone, free 10074/DON combination, PS/10074 or PS/10074-DON at a 10074 dosage of 10 mg/kg. (**C-E**) The relative abundance of CD4^+^ and CD8^+^ T cells in tumor tissues. (**F-H**) Flow cytometry gating and histograms of macrophages and CD11b^+^/Gr-1^+^ MDSCs in mouse tumors. The results are expressed as the mean ± SEM of measurements from 4 animals per group, and each dot represents a measurement from one animal. *p < 0.05, **p < 0.01, ***p < 0.001).

The immune cell populations in the tumor tissues were also examined in the Myc-CaP model (**Figure 10**). Tumors from the mice treated with PS/10074-DON exhibited significant increases in CD45^+^ cells and CD4^+^ and CD8^+^ T cells (**Figure 10A-E**), along with a decrease in MDSCs (**Figure 10F and H**). Overall, the data from both tumor models suggest an improvement in the tumor immune microenvironment in the tumors treated with PS/10074-DON.

## Discussion

The successful targeting of MYC in the clinic is challenging and has not been achieved to date. Since MYC is a universal amplifier of numerous target genes implicated in metabolism, proliferation, differentiation, and apoptosis 4,40, both MYC hyperactivation and repression could result in enormous transcriptional and translational perturbations. Many groups have reported a beneficial effect in cancer treatment by blocking Myc overactivity directly or indirectly 8,37,39. However, few studies have reported on the potential concerns of blocking MYC transcriptional activities.

**Figure 11:**
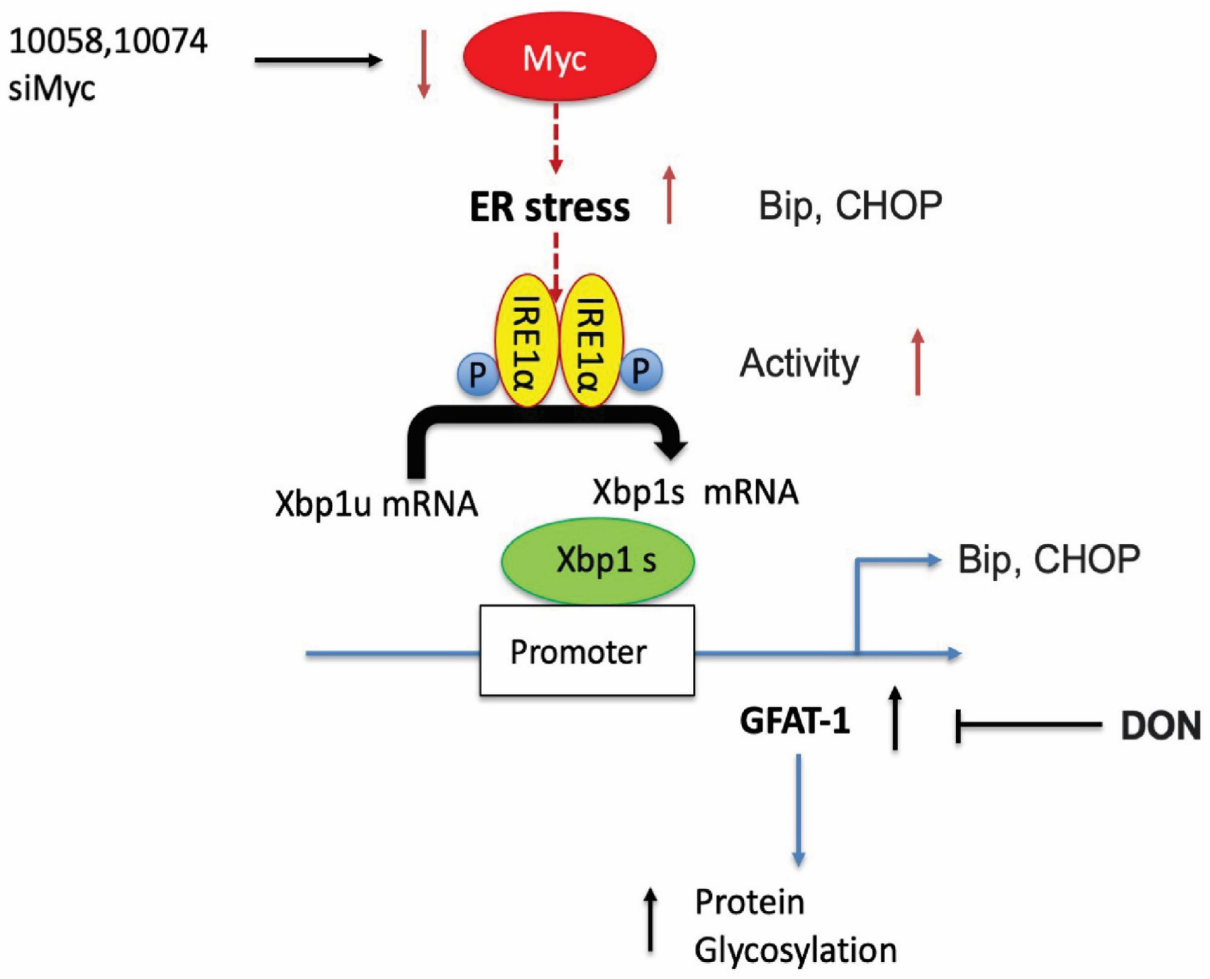
Hypothesized mechanisms of Myc inhibitors modulating GFAT1 expression.

Our study thus far reveals that both pharmacological and genetic perturbation of MYC activity leads to upregulation of the metabolic enzyme GFAT1 and enhanced glycosylation in prostate cancer models (**Figure 1-2**). These changes appear to be attributed to the induction of ER stress and mediated by the IRE1α/XBP1 pathway as a result of Myc transcriptional inhibition (**Figure 4-5**). All these data fit into our proposed mechanism of how inhibition of c-Myc activity induces GFAT1 upregulation and increased O-GlcNAcylation (**Figure 11**).

Our work raises questions about the role of MYC deregulation in metabolic reprogramming. MYC is involved in ER stress regulation via multiple mechanisms 8,42. MYC was reported to directly bind to and regulate the IRE1 promoter and enhancer regions in a breast cancer model 28. Paradoxically, our data suggested that inhibition of MYC also induced ER stress and activated the IRE1α/XBP1 pathway (**Figure 4**). The inconsistency is probably due to different cancer models or cellular contexts. On the other hand, MYC and MYC target genes are involved in a large signaling network with a highly complicated and adaptive nature, affecting both proliferation and apoptosis depending on the tissue type and the context 1,9. There is evidence suggesting that inhibition of MYC could also cause perturbation of ER homeostasis or facilitate the activation of the unfolded protein response (UPR) 8,43. Our findings could be explained as a feedback mechanism through which tumor cells adapt to MYC suppression and evolve to use alternative signaling pathways to support sustained proliferation.

The role of hyper-O-GlcNAcylation in promoting tumor progression has been well studied. O-GlcNAcylation not only directly modulates the activity of many oncogenes, such as p53, NFκB, and MYC 11,44,46, but also indirectly affects the self-renewal potential and metastatic ability of tumor cells by altering immune checkpoint molecules and impacting tumor microenvironments 13,15,47,48. Various molecules are involved in protein glycosylation, including GFAT1, OGT and other enzymes. Interestingly, Myc inhibition led to selective upregulation of GFAT-1 with no effect on the level of OGT in the 3 prostate cancer cell lines tested in our study. A pro-survival role of GFAT-1 following Myc inhibition was clearly demonstrated by the data that both pharmacological inhibition of GFAT via DON and GFAT-1 knockdown via RNAi led to sensitization of tumor cells to Myc inhibitors (**Figure 4**). The role of GFAT-1 in the synergistic effect was further demonstrated by the data showing that knockdown of GFAT-1 significantly attenuated the response of tumor cells to 10074/DON combination treatment (**Figure 4**). Our study pointed out for the first time that MYC-targeting compounds could potentially increase the O-GlcNAcylation level in cancer cells and compromise the antitumor effect of MYC inhibition. Our data also suggest the potential of combining Myc and GFAT-1 inhibitors as a novel approach for the treatment of prostate cancer.

One major challenge in translating Myc inhibition into an effective therapy is the limited bioavailability of Myc inhibitors. We developed a simple PS-based nanocarrier to improve the delivery of Myc inhibitors. To facilitate codelivery of 10074 and DON, we also developed a prodrug conjugate of 10074 and DON that could be effectively loaded into PS nanocarriers. Delivery of 10074-DON via PS led to significant improvement in antitumor activity. This treatment was also associated with an improvement in the tumor immune microenvironment, as shown by increases in CD4^+^ and CD8^+^ T cells and a decrease in MDSCs (**Figure 9-10**). Targeting Myc in cancer cells has been reported to improve the antitumor immune response 37,47,49. In our study, no significant changes were seen in immune cell populations with either PS/10074 or the free 10074+DON combination. This might be due to the differences in the treatment regimen and/or the tumor types involved. More studies are needed in the future to better define the underlying mechanisms.

In summary, our study suggests that inhibition of Myc transcriptional activity leads to upregulation of GFAT1 and increased O-GlcNAcylation in prostate cancer models. GFAT-1 induction may be attributed to ER stress as a result of Myc inhibition and is mediated by the IRE1a/XBP-1s/GFAT-1 pathway. Codelivery of 10074 and DON via a PS nanocarrier led to enhanced antitumor activity along with an improvement in the tumor immune microenvironment. With an improved understanding of how MYC deregulation triggers an adapted or feedback mechanism in cancer cell metabolic signaling, our findings may provide new mechanistic insights and alternative therapeutic options for improved cancer treatment.

## Materials and Methods

### Materials

10058-F4 and 10074-G5 were purchased from MedChemExpress (MCE) (Monmouth Junction, NJ). Laminarin and succinate anhydride were purchased from TCI America (Portland, OR). DON was purchased from Bachem (Torrance, CA). All other chemicals were purchased from Sigma-Aldrich (St. Louis, MO) and used without further purification unless otherwise noted.

### Synthesis of 10074-derivatized laminarin

The 10074-laminarin conjugate was synthesized following the synthesis route depicted in **Scheme 1**.

*Synthesis of** compound 1***: Laminarin (1 g), succinate anhydride (400 mg) and DMAP (50 mg) were dissolved in DMF (10 mL). The solution was gently shaken at 37 °C for 24 h. Cold ether was then added to the reaction mixture, and the precipitate was collected and washed with cold ether 3 times. The solid was dried in an oven at 60 °C for 12 h.

*Synthesis of **compound 2***: Compound 1 (1 g), EDCI (300 mg), HOBt (200 mg), and 10074-G5 (100 mg) were dissolved in DMF (10 mL). The solution was gently shaken at 37 °C for 24 h. Cold ethanol was then added to the reaction mixture, and the light red precipitate was collected and washed with cold ethanol 3 times. The solid was dried in an oven at 60 °C for 12 h.

*Synthesis of** compound 3***: Compound 2 (1 g), EDCI (300 mg), HOBt (200 mg), and PEG2K-NH2 (200 mg) were dissolved in DMF (10 mL). The solution was gently shaken at 37°C for 24 h. Then, decylamine (100 mg), EDCI (300 mg), and HOBt (200 mg) were added to the solution, and the reaction was allowed to proceed at 37°C for another 24 h. Cold ethanol was added to the reaction mixture, and the light red precipitate was collected and washed with cold ethanol 3 times. The solid was dissolved in water and dialyzed against water for 48 h. The final product was collected and freeze-dried. The ^1^H-NMR spectrum of the synthesized polymer was examined on a Varian-400 FT-NMR spectrometer at 400.0 MHz with DMSO‑d6 as the solvent.

**Synthesis of 10074-DON**: The 10074-DON prodrug conjugate was synthesized following the synthesis route depicted in **Scheme 2**.

*Synthesis of **compound 2***: 10074-G5 (332 mg, 1 mmol), compound **1** (348 mg, 1.5 mmol), DCC (310 mg, 1.5 mmol), and DMAP (20 mg, 0.16 mmol) were dissolved in 10 mL DMF. The solution was shaken at 37 °C for 24 h. Cold ether was then added to the reaction mixture, and the precipitate was collected and washed with cold ether 3 times. The solid was dissolved in a TFA/CH_2_Cl_2_ (V/V = 1/1) solution (10 mL). The solution was gently shaken at room temperature for 2 h followed by rotary evaporation to dryness. The solid was then purified by silica gel column with ethanol/CH_2_Cl_2_ (V/V = 1/9) as eluent. Approximately 200 mg of compound **2** was obtained with a yield of 45%.

*Synthesis of **compound 10074-G5-DON***: Compound **2** (100 mg, 0.22 mmol) and DON (38 mg, 0.22 mmol) were mixed in 5 mL CH_2_Cl_2_. The mixture was left to stand for 10 minutes at room temperature followed by rotary evaporation to obtain the final product with quantitative yield.

### Preparation and characterization of 10074-DON-loaded PS nanoparticles

Blank and 10074-G5-DON prodrug-loaded micelles were prepared by the film hydration method. The 10074-G5-DON prodrug solution (5 mg/mL) was prepared by dissolving in dichloromethane/methanol (1:1, v/v). Then, the prodrug solution was mixed with 10074 polymer (5 mg/mL in dichloromethane) at different polymer/drug weight ratios. The solvent was removed by air flow, followed by 2 h in vacuum to further remove the remaining solvent. The thin film formed was hydrated with saline. The size distribution of micelles was measured via dynamic light scattering (DLS). The 10074 prodrug concentrations in micelles were determined by a Waters Alliance 2695 Separations Module combined with a Waters 2489 UV/Vis Detector (UV wavelength 470 nm). The drug loading capacity (DLC) and drug loading efficiency (DLE) of 10074 prodrug were calculated according to the following equations:

DLC (%) = [weight of loaded drug/(weight of polymer + input drug)] × 100%

DLE (%) = (weight of loaded drug/weight of input drug) × 100%

### Cell Culture

The PC-3 human prostate cancer cell line and the RM1 and Myc-CaP murine prostate cancer cell lines were purchased from ATCC (Manassas, VA). The PC3 cell line was maintained in complete RPMI-1640 medium (Gibco, Gaithersburg, MD, USA) supplemented with 10% fetal bovine serum (FBS) and 1% penicillin/streptomycin. The RM-1 mouse cell line and Myc-Cap cell line were maintained in DMEM (Gibco, Gaithersburg, MD, USA) supplemented with 10% fetal bovine serum (FBS) and 1% penicillin/streptomycin.

### Animals

Six-week-old male C57BL/6J (WT) mice and eight-week-old male NU/J mice were purchased from Jackson Laboratories (Bar Harbor, ME, USA). Animal maintenance and procedures were conducted according to a protocol approved by the Institutional Animal Care and Use Committee (IACUC) at the University of Pittsburgh.

For the *in vivo* therapeutic study, C57BL/6J mice were s.c. inoculated with RM-1 tumor cells (2×10^5^ cells per mouse). Mice were randomly grouped (N=6) when the tumor volume reached ~50 mm^3^ and treated with PBS, PS (nanocarrier), free 10074/DON combination (dissolved in ethanol/Cremophor), PS/10074 or PS/10074-DON (10 mg 10074/kg) once every 3 days 5 times via tail vein injection. Tumor sizes were measured every 2 days in two dimensions using a caliper, and the tumor volumes were calculated with the formula: V=(AxB^2^)/2 (A and B are the long and short diameters of the tumor). The relative tumor volume was calculated to compare the different treatment groups. Mice were sacrificed when the tumor volume reached ~2,000 mm^3^. A similar study was conducted with another murine prostate cancer model (Myc-Cap) in C57BL/6 mice. The effectiveness of PS/10074-DON was further evaluated in a human prostate cancer xenograft model (PC3) in NU/J mice.

### Quantitative real-time PCR

Total RNA was isolated from cell lysates or mouse tissues with TRIzol reagent (Invitrogen Life Technologies, Carlsbad, CA, USA). The expression levels of multiple mRNAs were detected by quantitative real-time PCR using the High-Capacity cDNA Reverse Transcription Kit (Thermo Fisher Scientific, Waltham, MA, US) and the LightCycler® 480 System (Roche, Indianapolis, IN, USA). The primers used for quantitative RT-PCR are shown in **Table S1**.

### Western Blot

Total proteins were extracted from cultured cells or mouse tumors lysed with radio-immunoprecipitation assay (RIPA) buffer (Thermo Fisher Scientific, Waltham, MA, USA) plus fresh protease and phosphatase inhibitors (Roche, Indianapolis, IN, USA). Proteins (20 μg) were loaded into each lane and separated by sodium dodecyl sulfate polyacrylamide gel electrophoresis. Proteins were then transferred to a 0.45 µm pore size nitrocellulose membrane (Bio-Rad, Hercules, CA, US), followed by immunodetection of target proteins using specific antibodies with chemiluminescent detection. The developed films were quantified with ImageJ software. The antibodies used in Western blotting are shown in **Table S2**.

### Immunofluorescence

Mouse tumors were embedded in an O.C.T. Compound. Seven-micron coronal sections were cut through the tumor with a cryostat. For immunofluorescence, tissue sections were rinsed with 0.1% TX-100 in PBS (PBST) three times, blocked with 1% goat serum and 2% BSA in PBS at room temperature for 1 h and then incubated with the primary antibody conjugated with fluorescence. Then, the slides were washed 3 times with PBST and mounted with Fluoromount-G™ mounting medium (Invitrogen™). Fluorescent images were captured with an All-in-One Fluorescence Microscope BZ-X800 (Keyence, Ōsaka, Japan).

### Flow study

The immune cell populations in the tumor tissues with various treatments were analyzed by flow cytometry. Cell suspensions from spleens or tumors were filtered, and red blood cells were lysed. For staining of cell surface molecules, cells were incubated with the indicated combinations of antibodies (CD11b, Gr-1, CD8, CD4, CD45, F4/80 and CD206). For intracellular staining, cells were fixed and permeabilized immediately after cell surface staining according to the manufacturer's description (eBioscience), followed by the addition of combinations of antibodies (FoxP3, IFN-γ and granzyme B) in permeabilization buffer. For IFN-γ staining, cells were stimulated with PMA (5 ng/ml) and ionomycin (500 ng/ml) in the presence of 10 mg/ml BFA for 4 h followed by cell surface and intracellular staining. All antibodies were purchased from BD Biosciences, and flow data were collected on an LSRFortessa (BD Biosciences). The data were analyzed using FlowJo software (Tree Star Inc.).

### Statistics

Data are presented as the mean ± SEM. Statistical analysis was performed with GraphPad Prism 6.0 software (GraphPad Software, San Diego, CA). One-way or two-way analysis of variance was adopted for analysis. Significance levels were denoted as *p <0.05, **p < 0.01, or ***p <0.001.

## Supplementary Material

Supplementary figures and tables.
