# Supplementary material for: Improved antitumor activity against prostate cancer via synergistic targeting of Myc and GFAT-1
Source: Theranostics. 2023 Jan 1;13(2):578–95. doi: 10.7150/thno.76614 (PMC9830436; doi:10.7150/thno.76614)
Supplement: Supplementary file 1 — Supplementary figures and tables. [file thnov13p0578s1.pdf]

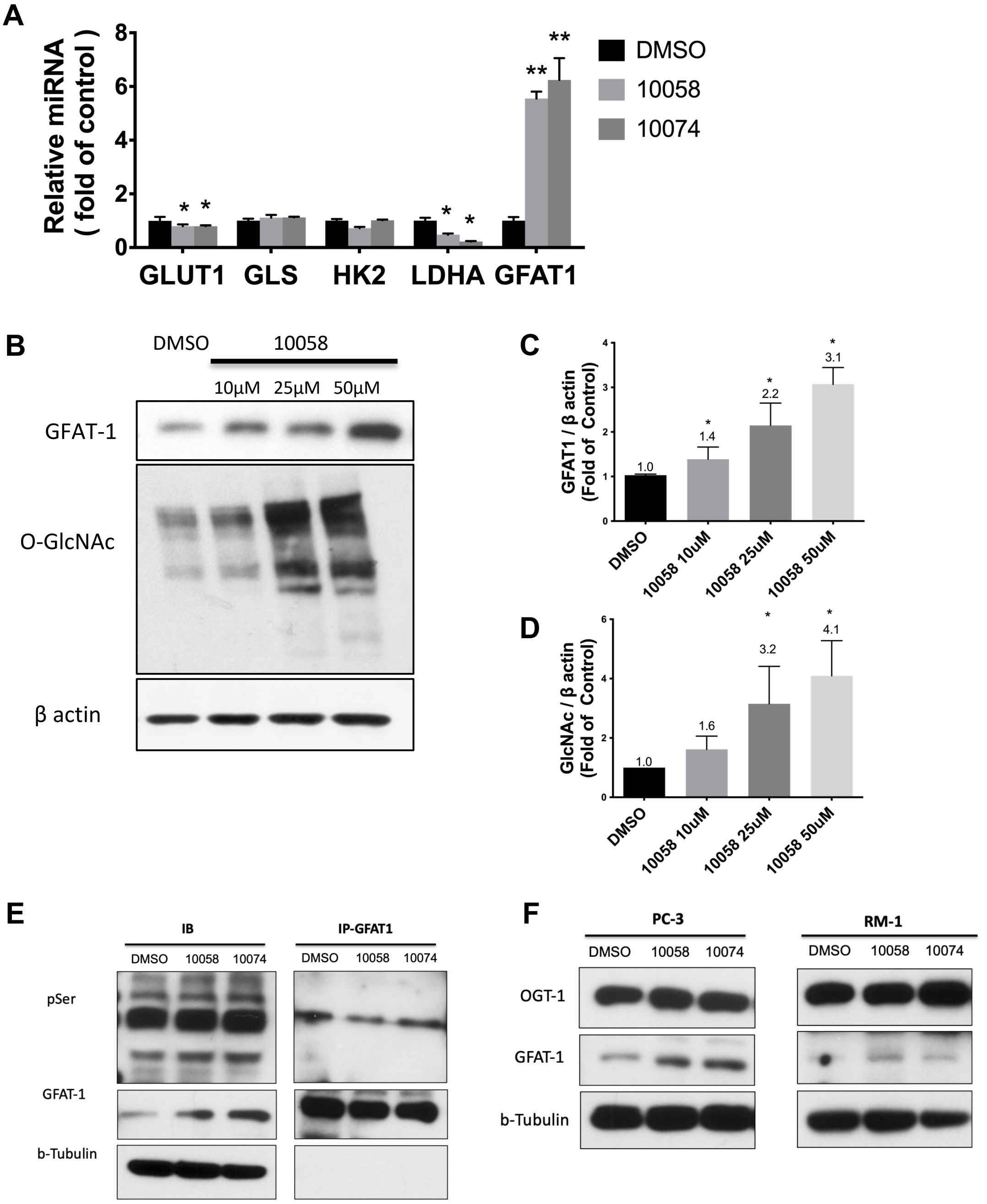

Figure S1. Inhibition of Myc leads to GFAT1 up-regulation and increased total protein glycosylation in PC3 cells in a dose dependent manner. (**A**) Real-time PCR analysis of pivotal genes involved in glucose metabolism in PC3 cells following treatment of Myc inhibitors. (**B-D**) Western blot assay revealed the effect of Myc inhibitor 10058 in up-regulating GFAT1 and total protein glycosylation in a dose dependent manner. (**E**) Immunoprecipitation of GFAT1 indicate that the phospho-GFAT1 was not altered by Myc inhibitors. (**F**) Western blot assay shows that 10058 and 10074 could not change OGT-1 level in prostate cell lines. The results are expressed as the mean ± SEM of triplicate measurements in each group. *p<0.05, **p<0.01, ***p<0.001.

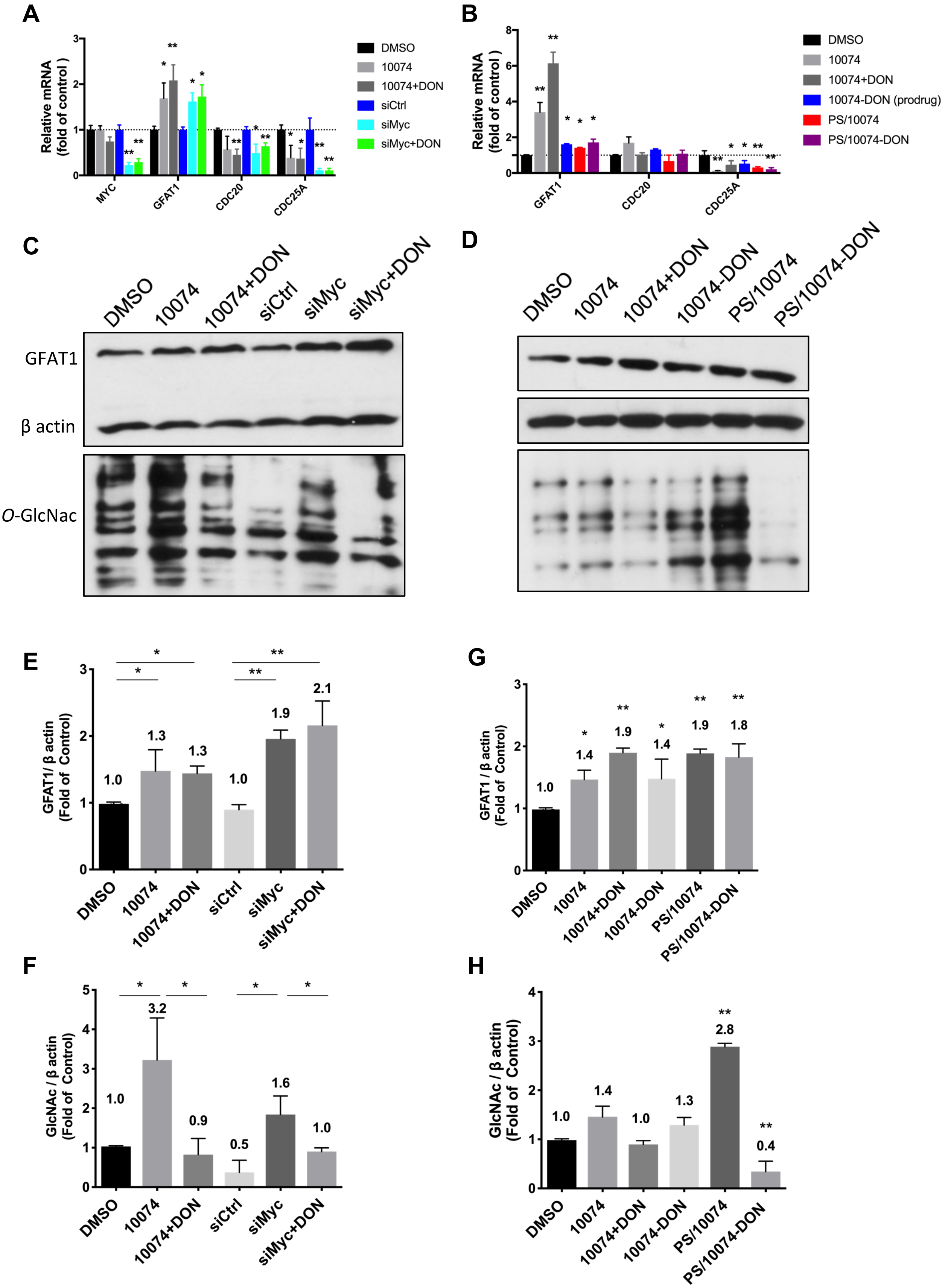

A
B
C
D
E
F
G
H
Relative mRNA (fold of control)
MYC
GFAT1
CDC20
CDC25A
DMSO
10074
10074+DON
siCtrl
siMyc
siMyc+DON
10074-DON (prodrug)
PS/10074
PS/10074-DON
10074-DON
β actin
O-GlcNac
GFAT1/ β actin (Fold of Control)
GFAT1 / β actin (Fold of Control)
GlcNAc / β actin (Fold of Control)

Figure S2. Inhibition of Myc leads to GFAT1 up-regulation and increased total protein glycosylation in Myc-CaP cell line: (**A-B**) Total RNAs in PC3 cells were isolated for qPCR analysis. (**A**) Myc-Cap cells were treated with 10074, 10074/DON, siMyc, or siMyc /DON combination. (**B**)The Myc-Cap cells were treated with 10074, 10074/DON combination, 10074-DON prodrug, PS/10074 or PS/10074-DON. (**C-D**) Total proteins in cell lysates were analyzed by Western blot. (**E-H**)Analysis of band intensity. The results are expressed as the mean ± SEM of triplicate measurements in each group. *p<0.05, **p<0.01, ***p<0.001.

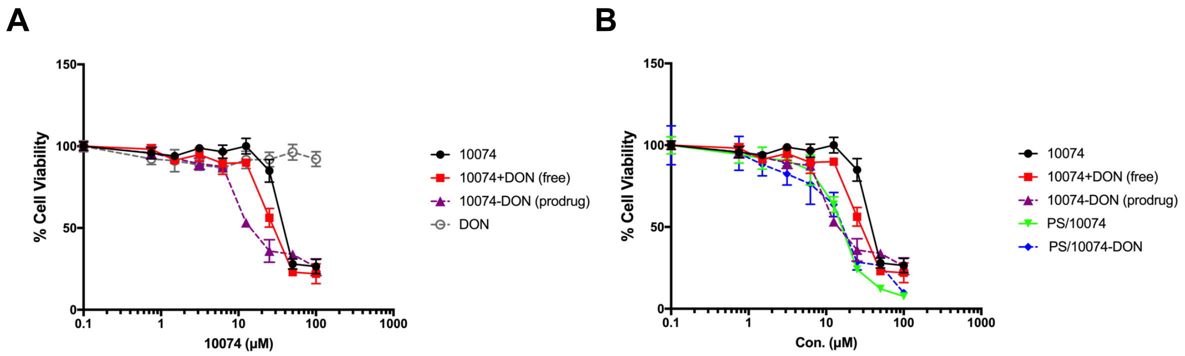

Figure S3. Cell viability of Myc-Cap cells receiving various treatments. (**A-B**) MTT assay of 10074 or 10074-DON in Myc-Cap cells treated with prodrug or carrier-loaded drug. The results are expressed as the mean ± SEM of triplicate measurements in each group. *p<0.05, **p<0.01, ***p<0.001.

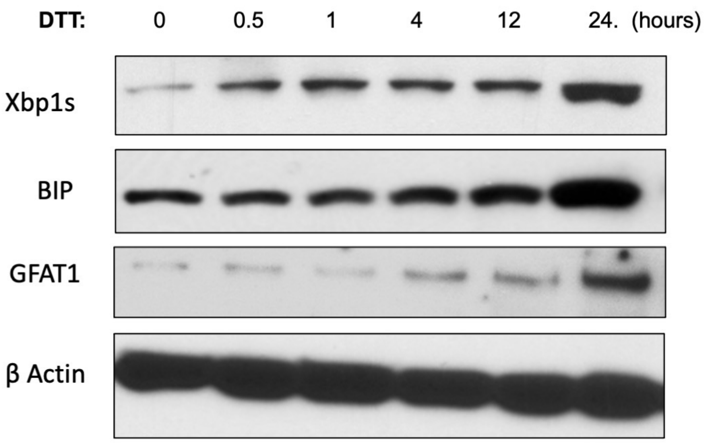

Figure S4. ER stress inducer DTT significantly induce Xbp1s, BIP and GFAT1 protein expression in a timely-dependent manner.

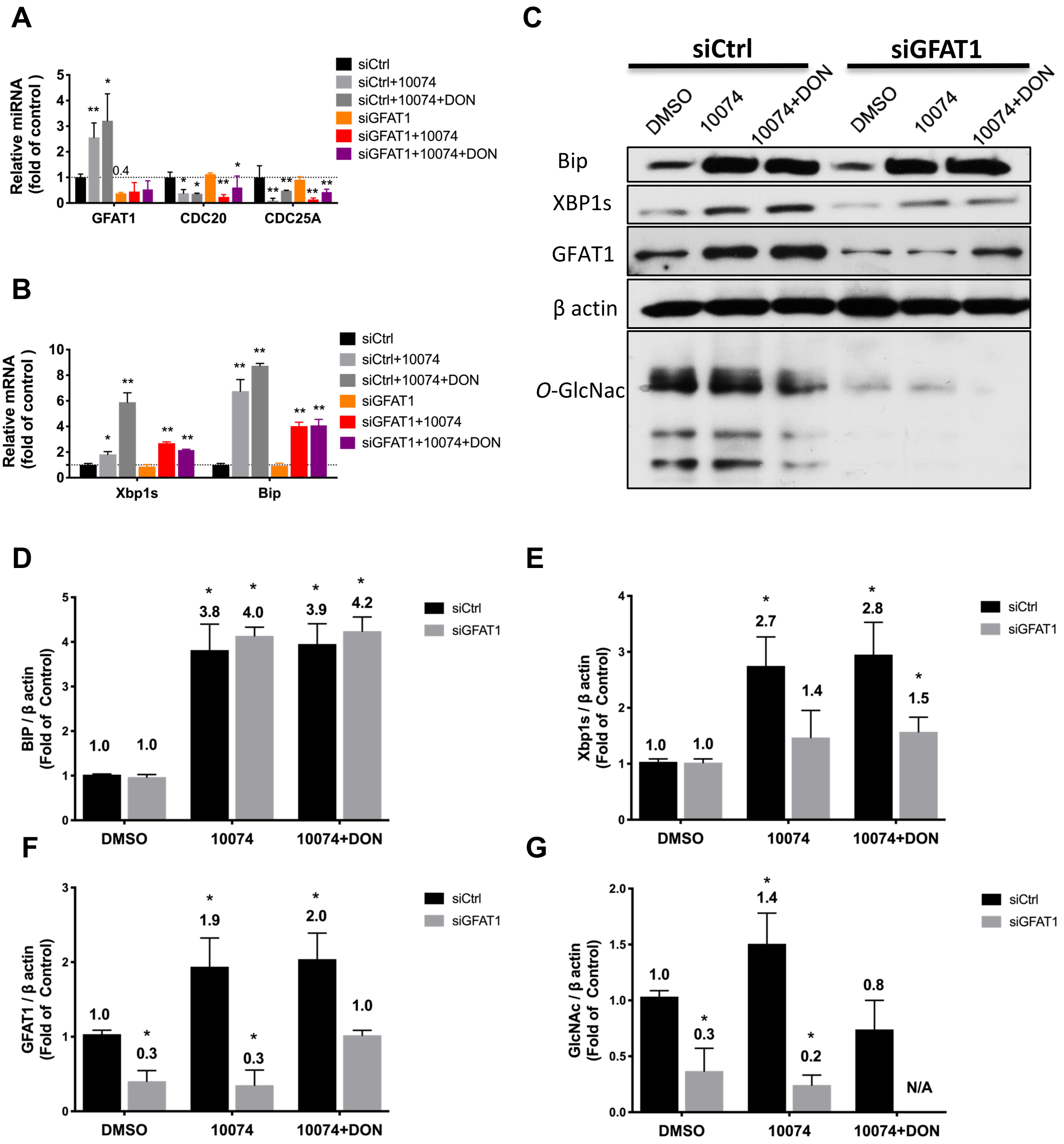

Figure S5. Knockdown of GFAT1 by siRNA attenuated 10074-induced protein glycosylation but did not affect the expression of Myc target genes as well as the expression of XBP1s and Bip: (**A-B**) Knockdown GFAT1 by siRNA abolished the effect of 10074 in up-regulating the expression of GFAT1 mRNA but did not affect the mRNA expression of other Myc target genes (**A**) as well as xbp1s and BIP (**B**). (**C**) Pre-transfection of GFAT-1 siRNA (siGFAT1) attenuated the effect of 10074 in up-regulating the protein level of GFAT1 and total protein glycosylation but did not affect the 10074-induced upregulation of XBP1s and BIP. (**D-G**) Quantification of western results.(The results are expressed as the mean ± SEM of triplicate measurements in each group. *p<0.05, **p<0.01.

A

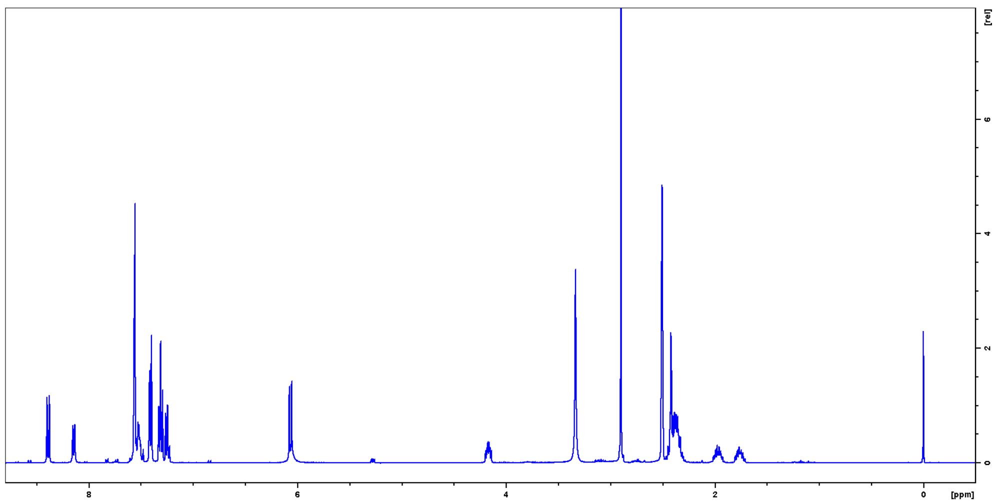

B

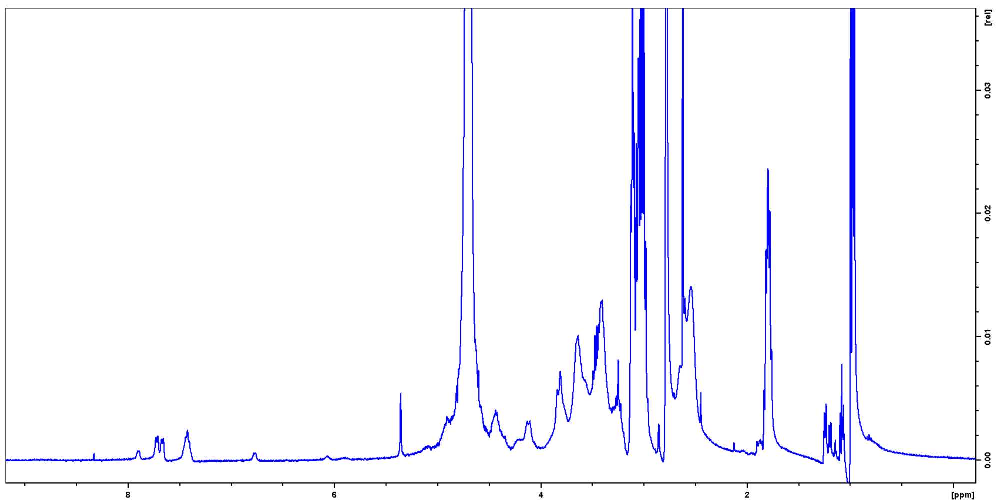

Figure S6. NMR spectrum of 10074-DON conjugate(A) and NMR spectrum of 10074-derivatized laminarin(B).

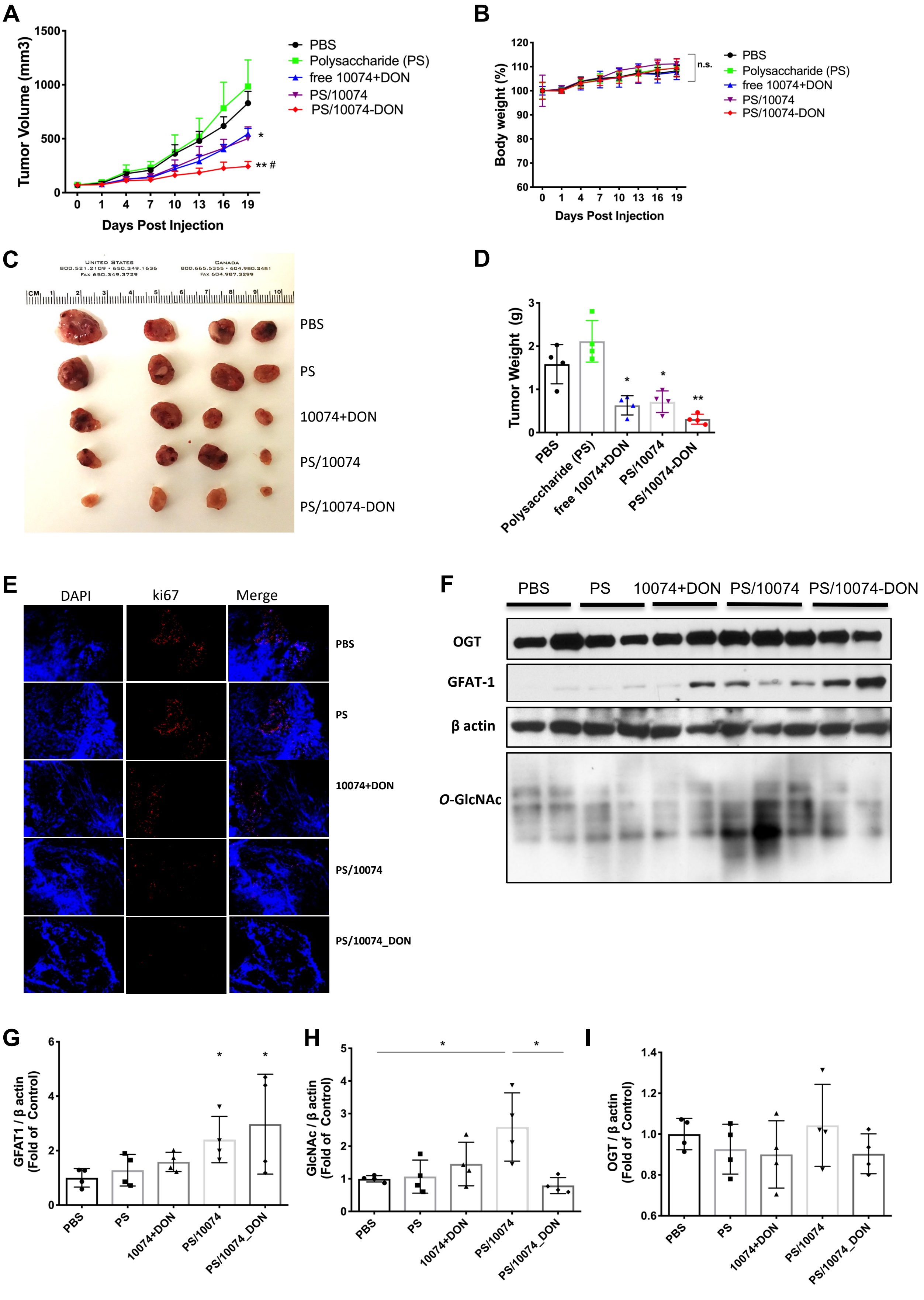

A
Tumor Volume (mm3)
1500
1000
500
0
0 1 4 7 10 13 16 19
Days Post Injection
PBS
Polysaccharide (PS)
free 10074+DON
PS/10074
PS/10074-DON
*
** #
B
Body weight (%)
120
110
100
90
80
70
60
0 1 4 7 10 13 16 19
Days Post Injection
n.s.
PBS
Polysaccharide (PS)
free 10074+DON
PS/10074
PS/10074-DON
C
PBS
PS
10074+DON
PS/10074
PS/10074-DON
D
Tumor Weight (g)
3
2
1
0
PBS
Polysaccharide (PS)
free 10074+DON
PS/10074
PS/10074-DON
*
*
**
E
DAPI
ki67
Merge
PBS
PS
10074+DON
PS/10074
PS/10074_DON
F
PBS
PS
10074+DON
PS/10074
PS/10074-DON
OGT
GFAT-1
β actin
O-GlcNAc
G
GFAT1 / β actin
(Fold of Control)
6
4
2
0
PBS
PS
10074+DON
PS/10074
PS/10074_DON
*
*
H
GlcNAc / β actin
(Fold of Control)
5
4
3
2
1
0
PBS
PS
10074+DON
PS/10074
PS/10074_DON
*
*
I
OGT / β actin
(Fold of Control)
1.4
1.2
1.0
0.8
0.6
PBS
PS
10074+DON
PS/10074
PS/10074_DON

Figure S7. *In vivo* anti-tumor effect of 10074-DON-loaded PS NPs in Myc-Cap tumor model: (**A**) Tumor growth curves in Myc-Cap tumor model. (**B**) Body weight changes during the treatment. (**C**) Gross images of the endpoint tumors. (**D**) Endpoint tumor weights in RM-1 tumor model. (**E**) Ki67 immunostaining of tumor sections. (**F**) Western blot analysis of the tumor tissues collected in (**C**) and the quantification by ImageJ (**G-I**). The results are expressed as the mean ± SEM of measurements from 6 animals per group, and each dot represents a measurement from one animal. *p<0.05, **p<0.01, ***p<0.001.

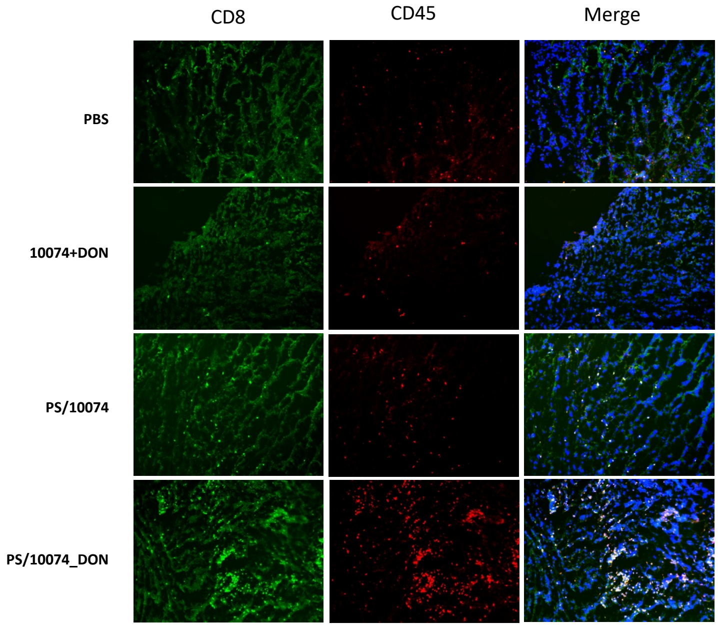

Figure S8. Immunostaining of infiltrating immune cells in tumor tissues with various treatments: RM-1 tumor-bearing mice received various treatments as described in **Fig. 8**. Infiltration of $CD4^+$ and $CD8^+$ T cells in tumor tissues was examined by staining of tumor sections with the respective specific antibody.

Table S1. Primers used in real-time PCR.

Primer	Species	Sequence
GAPDH-F	human	GGTGAAGGTCGGAGTCAACG
GAPDH-R	human	TGGGTGGAATCATATTGGAACA
MYC-F	human	AATGAAAAGGCCCCCAAGGTAG
MYC-R	human	GTCGTTTCCGCAACAAGTCCT
CDC20-F	human	CGCTATATCCCCCATCGCAG
CDC20-R	human	CTGATAACCCTCTGGCGCAT
CDC25A-F	human	AGACCTGTATCTCGTGGCTG
CDC25A-R	human	CATTTGAGGAAAGCATCCGAGC
CDC45-F	human	GTGATTTGGCGGGAGTCTTG
CDC45-R	human	CGAAGAGAAGGACCCTCTGG
GFAT1-F	human	AGCAGTGCAAACCCTCCAGATGG
GFAT1-R	human	TGAACCCCACAATCTGTCTCCCG
GLUT1_F	human	TTGCAGGCTTCTCCAACTGGAC
GLUT1_R	human	CAGAACCAGGAGCACAGTGAAG
GLS_F	human	CAGAAGGCACAGACATGGTTGG
GLS_R	human	GGCAGAAACCACCATTAGCCAG
LDHA_F	human	GGATCTCCAACATGGCAGCCTT
LDHA_R	human	AGACGGCTTTCTCCCTCTTGCT
HK2_F	human	GAGTTTGACCTGGATGTGGTTGC
HK2_R	human	CCTCCATGTAGCAGGCATTGCT
XBP1u_F	human	CAGACTACGTGCACCTCTGC
XBP1u_R	human	CTGGGTCCAAGTTGTCCAGAAT
GFAT1-F	mouse	GGA ATC ATC ACC AAC TAC AAA GAC
GFAT1-R	mouse	AAT ACT CCA CTG CTT TTT CTT CCA C
XBP1u_F	mouse	CAGACTACGTGCACCTCTGC
XBP1u_R	mouse	CAGGGTCCAACTTGTCCAGAAT
XBP1s_F	mouse	GCTGAGTCCGCAGCAGGT
XBP1s_R	mouse	CAGGGTCCAACTTGTCCAGAAT
CHOP_F	mouse	AAGATGAGCGGGTGGCAGCG
CHOP_R	mouse	GCACGTGGACCAGGTTCTGCT
GRP78_F	mouse	TGCAGCAGGACATCAAGTTC
GRP78_R	mouse	TACGCCTCAGCAGTCTCCTT

Table S2. Primary antibodies used in western blot.

	Species	Dilution	Cat.#	Brand
**GFAT1**	Rabbit	1:1000	3818S	Cell Signaling Technology (Danvers, MA,US)
**O-GlcNac**	Mouse	1:500	ab201995	Abcam (Cambridge, UK)
**c-MYC**	Rabbit	1:1000	18583S	Cell Signaling Technology (Danvers, MA,US)
**β Actin**	Rabbit	1:4000	4970S	Cell Signaling Technology (Danvers, MA,US)
**Xbp1s**	Rabbit	1:1000	24868-1-AP	Thermo Fisher Scientific (Waltham, MA, US)
**IRE1α**	Rabbit	1:1000	3294S	Cell Signaling Technology (Danvers, MA,US)
**pIRE1α**	Rabbit	1:1000	PA1-16927	Thermo Fisher Scientific (Waltham, MA, US)
**BIP**	Rabbit	1:1000	3177S	Cell Signaling Technology (Danvers, MA,US)
**OGT**	Rabbit	1:1000	24083S	Cell Signaling Technology (Danvers, MA,US)
